# Biotechnologically potential genes in a polysaccharide-degrading epibiont of the Indonesian brown algae *Hydroclathrus* sp.

**DOI:** 10.1186/s43141-023-00461-5

**Published:** 2023-02-14

**Authors:** Stalis Norma Ethica, Dewi Seswita Zilda, Oedjijono Oedjijono, Muhtadi Muhtadi, Gintung Patantis, Sri Darmawati, Sri Sinto Dewi, Agus Sabdono, Agustinus Robert Uria

**Affiliations:** 1 Magister Program of Clinical Laboratory Science, Universitas Muhammadiyah Semarang (UNIMUS), Jalan Kedungmundu Raya, Semarang, 50273 Indonesia; 2 Research Center for Deep Sea, Earth Sciences and Maritime Research Organization, National Research and Innovation Agency (BRIN), Jl. Pasir Putih Raya, Pademangan, North Jakarta City, Jakarta 14430 Indonesia; 3 Faculty of Biology, Universitas Jenderal Soedirman, Purwokerto, 53122 Indonesia; 4 Faculty of Pharmacy, Universitas Muhammadiyah Surakarta (UMS), Sukoharjo, 57162 Indonesia; 5 Research Center for Marine and Land Bioindustry, Earth Sciences and Maritime Research Organization, National Research and Innovation Agency (BRIN), Kodek Bay, North Lombok, West Nusa Tenggara 83352 Indonesia; 6 Diploma Study Program of Medical Laboratory Technology, Faculty of Nursing and Health Sciences, Universitas Muhammadiyah Semarang, Semarang, 50273 Indonesia; 7 Department of Marine Science, Faculty of Fisheries and Marine Science, Diponegoro University, Semarang, 50272 Indonesia; 8 Faculty of Pharmaceutical Sciences, Hokkaido University, Kita 12 Nishi 6 Kita-ku, Sapporo, 060-0812 Japan; 9 Global Station for Biosurfaces and Drug Discovery, Global Institution for Collaborative Research and Education (GI-CoRE), Hokkaido University, Kita 12 Nishi 6, Sapporo, 060-0812 Japan

**Keywords:** Marine macroalgae, Bacterial epibiont, Genome sequencing, Biotechnologically potential genes, Ecological role, Antimicrobial activity

## Abstract

**Background:**

Marine bacteria have recently attracted increasing attention to be harnessed for the production of valuable enzymes, vitamins, and bioactive compounds. Bacteria associated with the surfaces of marine macroalgae, called epibionts, are particularly interesting from ecological and biotechnological points of view, as they often exhibit antimicrobial activities to compete with pathogenic bacteria for nutrients and spaces. In search for biotechnologically potential genes from marine bacteria, we sequenced and analysed the genome of the epibiont HI03-3b, a polysaccharide-degrading bacterium associated with the surface of the Indonesian brown algae *Hydroclathrus* sp.

**Results:**

The algal epibiont HI03-3b has a genome of approximately 4,860,704 bp in size with 42.02 mol% G + C content, consisting of 5655 open reading frames (ORFs), 4409 genes coding for proteins (CDSs), 94 genes for tRNAs, and 32 genes for rRNAs. The genome sequence of HI03-3b was most closely related to that of *Cytobacillus firmus* NCTC10335 with the average amino acid identity (AAI) of 95.0 %, average nucleotide identity (ANI) of 94.1 %, and a recommended DNA-DNA hybridization (DDH) of 57.60 %. These scores are lower than the most frequently used standard for species demarcation (95% ANI cutoff) and the new species threshold (DDH > 70.0% for the same bacterial species). Some differences in genome features and gene composition were observed between HI03-3b and NCTC10335, such as genes encoding carbohydrate active enzymes. These suggest that HI03-3b is unique and likely a novel species within *Cytobacillus* genus, and we therefore proposed its name as *Cytobacillus wakatobiense* HI03-3b. Genome sequence analyses indicated the presence of genes involved not only in polysaccharide and protein degradation but also in vitamin and secondary metabolite biosynthesis. Some of them encode enzymes and compounds with biotechnological interest, such as protease, chitinase, subtilisin, pullulanase, and bacillolysin, which are often associated with antimicrobial or antibiofilm activities. This antimicrobial potential is supported by our finding that the extracellular protein fraction of this epibiont inhibited the growth of the bacterial pathogen *Staphylococcus aureus*.

**Conclusion:**

The epibiont *Cytobacillus* HI03-3b harbours genes for polysaccharide and protein degradation as well as for natural product biosynthesis, suggesting its potential ecological roles in outcompeting other bacteria during biofilm formation as well as in protecting its algal host from predation. Due to the presence of genes for vitamin biosynthesis, it might also provide the algal host with vitamins for growth and development. Some of these metabolic genes are biotechnologically important, as they could become a platform for bioengineering to generate various seaweed-derived substances sustainably, such as antibiofilm agents and vitamins, which are beneficial for human health.

**Supplementary Information:**

The online version contains supplementary material available at 10.1186/s43141-023-00461-5.

## Background

Marine macroalgae, also called seaweeds, are valuable sources of health-promoting secondary metabolites, enzymes, and vitamins for being developed as nutraceuticals, pharmaceuticals, and cosmeceuticals [24, 36, 42]. Particularly, enzymes from seaweeds have attracted increasing attention for biotechnological applications due to their unique features [55]. However, the natural purification of algae-derived enzymes is extremely difficult due to their high content of polysaccharides, polyphenols, and stable cell walls [45]. Furthermore, the limited supply of seaweed-derived bioactive compounds and enzymes represents a big challenge in their development into high-value marketable products, because most existing cultivation techniques developed for producing commoditized biomass may not necessarily be optimized for seaweed bioactive production [24].

Microorganisms associated with seaweeds have recently been recognized as the producers of novel bioactive compounds and enzymes [40]. Their ability to produce bioactives is considered as an ecological strategy to compete for nutrients and space on the surfaces of marine macroalgae [18]. This strategy also helps their algal hosts to chemically defend against the secondary colonization by other microscopic and macroscopic epibiota [17]. A notable example of epibionts that play this crucial ecological role is *Pseudoalteromonas* species inhabiting seaweed surfaces, as they produce toxic compounds, bacteriolytic substances, and extracellular enzymes for outcompeting other bacteria during biofilm formation [26, 27]. Continuous attempts to isolate potential seaweed-associated bacteria for identifying biotechnologically relevant genes are urgently needed to produce bioactive compounds and enzymes with biotechnological interest in sustainable ways.

In search for biotechnologically potential genes from seaweed epibionts, we initially isolated a novel polysaccharide-degrading bacterial species associated with the Indonesian brown algae *Hydroclathrus* sp. [70]. We found that the cell-free culture of this epibiont was able to inhibit the bacterial pathogen *S. aureus*, indicating its potential ability to produce antimicrobial substance extracellularly. This preliminary result encouraged us to sequence the whole genome of this bacterium in order to better understand its ecological role and biotechnological potential. Analysing the HI03-3b genome sequence has enabled us to identify metabolic genes, including those involved in polysaccharide and protein degradation as well as in natural product biosynthesis. Since polysaccharides and proteins represent key components of the extracellular polymeric substances (EPS) of pathogenic microbial biofilms [57], this finding could become a basis for further exploring antimicrobial enzymes and compounds to treat persistent pathogenic biofilms. Subsequent heterologous expression of these genes is necessary to produce useful enzymes or compounds in sustainable ways for biotechnological applications.

## Methods

### Isolation and bioassay of a potential algae-associated epibiont

**Fig. 1:**
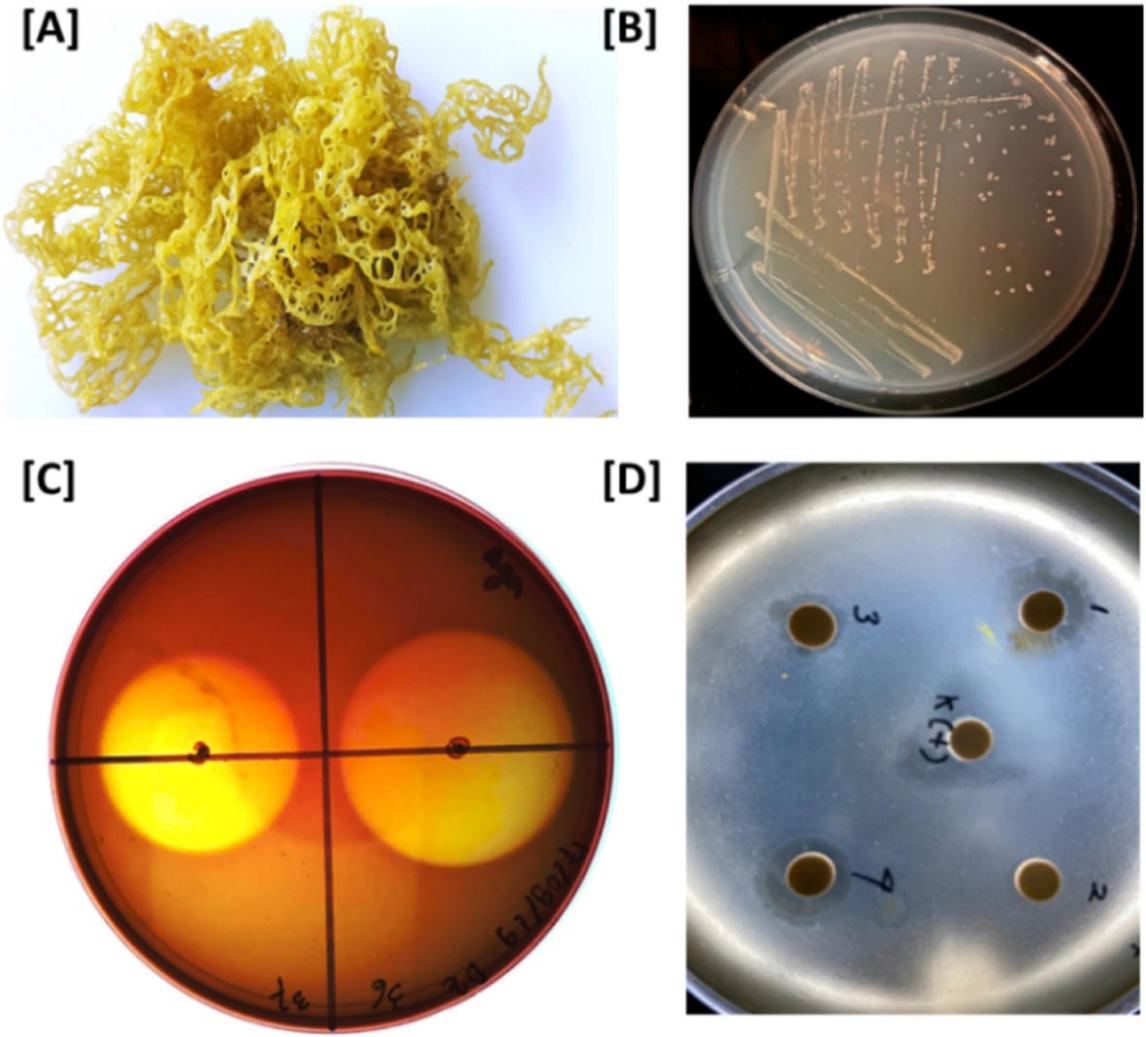
Isolation of the potential algae-associated epibiont HI03-3b. The Indonesian brown algae *Hydroclathrus* sp. (photo taken by S. N. Ethica) where HI03-3b was isolated (**A**) and colony morphology of HI03-3b (**B**). Bioassays of HI03-3b showing a clearing zone surrounding colonies (duplo) on an alginate-containing MS plate after Lugol staining (**C**) and inhibition zone by the extracellular culture against the lawn bacterial pathogen *S. aureus* (**D**)

A polysaccharide-degrading bacterium, designated as HI03-3b, was isolated from the brown algae *Hydroclathrus* sp. inhabiting sandy shallow water around Hoga Island, Wakatobi, South Sulawesi, Indonesia, at the site coordinate of 5.28317 S and 123.45377 E. Briefly, the algae sample was collected at the depth of 0.5–1.0 m (Fig. 1A). It was placed in a 50-ml conical tube and stored in a cool box. A total of 5 g seaweed sample was put into a bottle containing sterile seawater and vortexed for approximately 10 min to release the bacterial cells attached on the surface of *Hydroclathrus* sp. Aliquot of the seaweed mixture was inoculated into solid minimal seawater (MS) media by dilution (10^−5^–10^−8^) and then incubated for 3–7 days at 30 °C. The MS medium was made up of 0.5% tryptone, 0.1% yeast extract, 0.1% sodium alginate, and 2% agar dissolved in seawater (30 ppt). The colonies growing on solid MS were isolated and purified. The ability of the bacterial isolates to degrade polysaccharides was assessed by a 24-h incubation and subsequent staining with Lugol’s iodine solution, followed by staining with CaCl_2_ solution (10%) with 1-h incubation to observe the capability of degrading alginate [70]. The most potential bacterial isolate was subsequently tested for the antimicrobial ability of its extracellular fraction against *Staphylococcus aureus* ATCC®25923™, an opportunistic bacterial pathogen that can cause various infections and food poisoning [14, 63]. Briefly, the cell-free culture was initially prepared and concentrated through a 10-kDa membrane filtration (Whatman). The concentrated extracellular fraction (50 μl) was applied on a paper disc placed on the solid medium streaked with *S. aureus*.

### Whole-genome sequencing

The genome of *Cytobacillus* sp. HI03-3b was sequenced using Oxford Nanopore Technology (ONT) carried out at the PT Genetika Science Indonesia, a certified service provider of ONT in Indonesia. Briefly, the HI03-3b genomic DNA was initially extracted using QIAGEN Genomic-tip 500/G followed by size selection with Agencourt AMPure XP beads and Circulomics Short Read Eliminator Kit. The libraries of HI03-3b genomic DNA for sequencing were subsequently constructed using Ligation Sequencing Kit (SQK-LSK109) according to the instructions of the manufacturer Oxford Nanopore Ltd., UK. In principle, the size-selected DNA fragments were end prepped using Ultra II End-prep enzyme mix and ligated with barcode adapter. With adapter-ligated DNA as the template, a barcoding PCR reaction was set up in LongAmp Taq 2× master mix. The resulting pooled barcoded libraries were then end/nick repaired and dA tailed using the NEBNext End Repair/dA-tailing module. The DNA concentration in each library preparation step was measured using Qubit fluorometer based on Qubit dsDNA HS (High Sensitivity) assay (Invitrogen™). The product of each reaction step was washed with 70% EtOH with the help of magnetic separator. The prepared library was loaded into the MinION™ Flow Cells on a ONT MinION sequencing device (Oxford Nanopore Ltd., UK). V14 kit chemistry in combination with the new R10.4.1 nanopore was used to provide Q20+ (≥ 99%) raw read accuracy with high sequencing yield. FastQC version 0.11.9 (written by Simon Andrews of Babraham Bioinformatics) was subsequently run to do control checks per base sequence quality data.

### Genome sequence assembly

Genome sequence assembly was carried out according to the main steps summarized in Fig. S1A. Bacterial genome sequence datasets were initially assembled using Flye Assembler Version 2.9, a de novo assembler for single-molecule sequencing reads [31, 56]. The Flye assemblies were subsequently polished with one-round Medaka (https://nanoporetech.com) for error correction to prepare high-quality genome sequences (available online: https://github.com/nanoporetech/medaka). Quality parameters of the assembled HI03-3b genome sequence using QUAST [22] (Galaxy Version 5.2.0+galaxy1). The parameters were set up with the minimum IDY% considered as proper alignment of 95.0 and the lower threshold for a contig length (in bp) of 500. The GC% content and read count of HI03-3b genomic sequence were determined using RSeQC (v 2.6.4) [67]. Taxonomic distribution analysis was conducted using MyTaxa Scan result from MiGA to determine the degree of affiliation or novelty of sequences based on the genome-aggregate average amino acid identity [38, 52]. The order and direction of contigs generated after genome sequence assembly were determined based on blastn pairwise alignment [2] and subsequently verified by average nucleotide identity (ANI) analysis [29] on PROKSEE with CGView Server [58, 59] using the complete genome sequences of closely related taxa as the references.

### Taxa novelty analysis

HI03-3b genomic sequence was subjected to MiGA (Microbial Genomes Atlas) [52] (version v1.0.0 — prima 14 April 2021) against all taxonomically classified taxa with available genome sequence data for determining its taxonomic classification (http://microbial-genomes.org/). This was based on average nucleotide identity and amino acid identity (ANI/AAI) concepts. ANI is a whole-genome similarity metric, which can facilitate high-resolution taxonomic analysis. In taxonomic studies, the standard for species demarcation is the 95% ANI cutoff [29]. HI03-3b genome sequence was compared to the genome sequences of closely related taxa using Mauve [13] (version snapshot 2015 February 25 build 0 (c) 2003–2015). Genome-to-Genome Distance Calculator (GGDC) 3.0 using a generalized linear model (GLM) [41] was run to confirm similarity level between the genome sequences of HI03-3b and the most closely related species. This analysis outcome was based on DNA-DNA hybridization (DDH) values to determine relatedness between bacterial species [21]. Phylogenetic analysis was conducted in MEGA X [32] based on the unweighted pair-group method with arithmetic mean (UPGMA) as the distance analysis method for constructing a tree [43].

### Genome sequence annotation

The assembled HI03-3b genome was visualized using PROKSEE on the CGView Server [58, 59] and subsequently annotated with Prokka version 1.1.0 [54], allowing the prediction of the numbers of CDSs (coding sequences) as well as genes for tRNAs and rRNAs (5S, 16S and 23S). These were verified using tRNAscan-SE 2.0 [9, 37] and NCBI record (Ref. Seq.: NZ_JAKDDU000000000.1). To predict genomic islands, the entire HI03-3b genome sequence was aligned against the complete genome sequence of the closely related taxon *Cytobacillus oceanisediminis* YPW-V2 [Accession Number: CP015506.1] using IslandViewer 4 [5] with the default parameters described in this link: www.pathogenomics.sfu.ca/islandviewer/about/. To predict HI03-3b primary metabolisms, all CDSs resulted from GeneMarkS analysis [6] were analysed using KofamKOALA [3] against KOfam, a customized HMM database of KEGG Orthologs (KOs) [3] combined with BLASTx analysis [2]. Carbohydrate-active enzyme (CAZy) database [8] was used as the reference to identify genes encoding carbohydrate active enzymes on HI03-3b genome sequence. By referring to gene position on contigs based on GeneMarkS analysis [6], biotechnologically potential genes were annotated on the circular HI03-3b genome map using PROKSEE on the CGView Server [58, 59]. Further analysis using AntiSMASH version 6.0 [7] was performed to identify natural product biosynthetic gene clusters (BGCs).

## Results

### Polysaccharide-degrading epibiont with antimicrobial activity

In search for biotechnologically potential genes from marine sources, we screened bacterial isolates from the surface of the Indonesian brown algae *Hydroclathrus* sp. (Fig. 1A). This led to the isolation of a polysaccharide-degrading bacterium, designated as HI03-3b, as indicated by the presence of a clearing zone around its colonies after staining with Lugol’s iodine solution and CaCl_2_ solution (10%) (Fig. 1B and C). Interestingly, the cell-free culture of this algae-associated epibiont exhibited inhibition against *S. aureus* (Fig. 1D), indicating its potential ability to produce antimicrobial protein or enzyme extracellularly. This subsequently encouraged us to sequence the genome of HI03-3b to identify useful genes that might encode proteins or enzymes with potential antimicrobial properties.

### Genome sequence and taxa novelty analyses

The FastQC analysis (written by Simon Andrews of Babraham Bioinformatics) indicated that the resulting HI03-3b sequencing data contained 1,097,444 sequence reads with the sequence length range of 139 to 14972 bp. All of these sequence reads were assembled into a circular genome of 4,860,704 bp with 42.02 mol% G + C content (Fig. S1B and C). The assembled HI03-3b genome sequence consisted of 11 contigs with the largest size of 2,127,197 bp. The assembly quality was checked using QUAST [22], showing good quality indicated by low values of L50 and L75. Taxonomic distribution analysis based on MyTaxa scan result from MiGA [52] showed the majority of light blue colour (Fig. S2), indicating the high quality of H03-3b genome sequence with minor contamination.

**Fig. 2:**
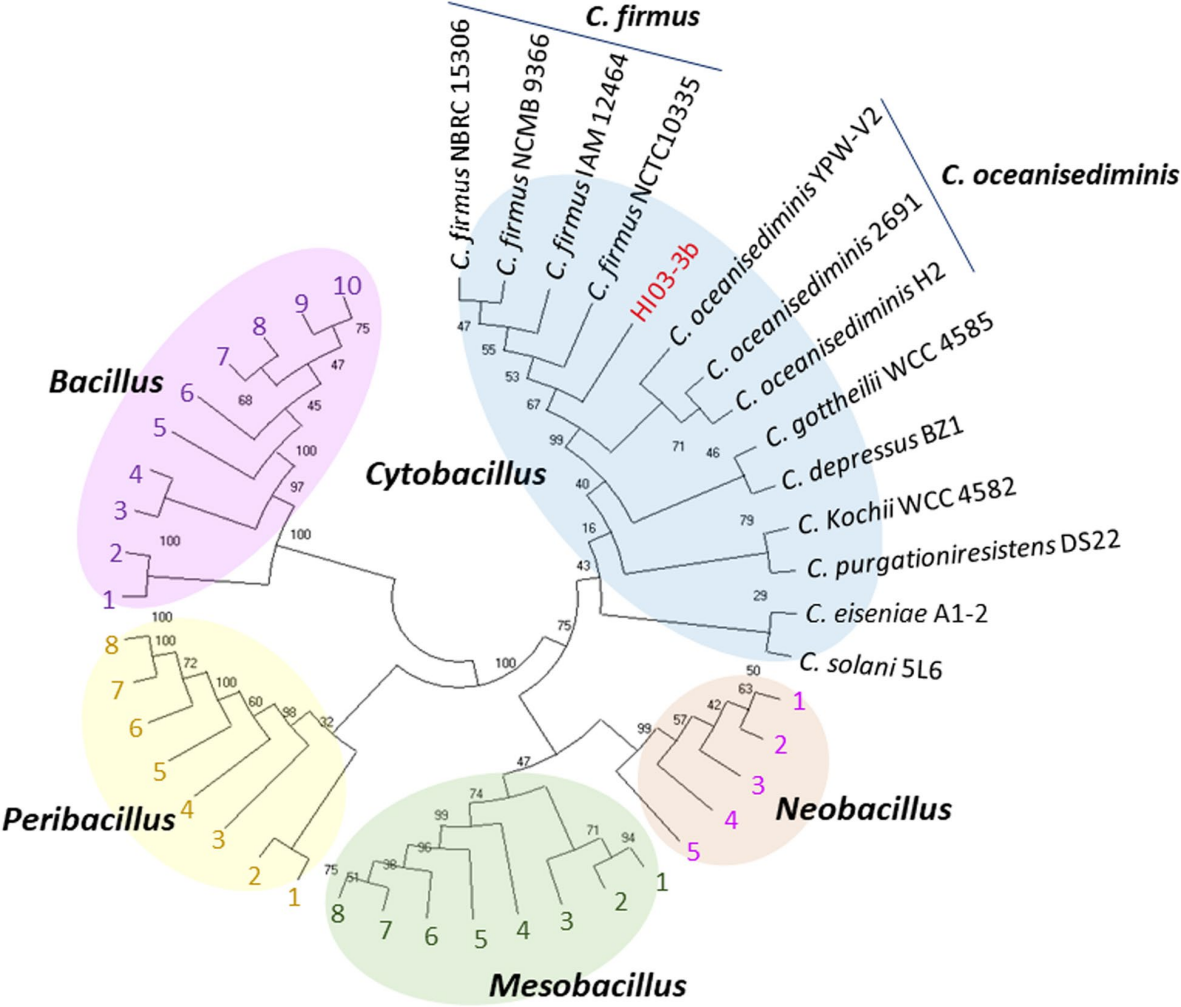
The phylogenetic analysis of HI03-3b 16S-rRNA gene sequence using the UPGMA method [43]. The bootstrap consensus tree inferred from 1000 replicates represents the evolutionary history of the taxa analyzed [19]. The evolutionary distances were calculated according to the maximum composite likelihood method [62]. Evolutionary analyses were performed in MEGA X [32], which involved 45 16S-rRNA gene sequences from representative members of some genera within Bacillaceae family. There were a total of 1593 positions in the final dataset. The phylogenetic tree covers representative members of some genera within Bacillaceae family (*Bacillus*, *Cytobacillus*, *Mesobacillus*, *Neobacillus*, and *Peribacillus*). *Neobacillus* clade is as follows: 1, *N. bataviensis* NBRC 102449; 2, *N. drentensis* IDA1967; 3, *N. novalis* NBRC 102450; 4, *N. niacini* NBRC 15566; and 5, *N. pocheonensis* Gsoil 420. *Mesobacillus* clade is as follows: 1, *M. foraminis* CV53; 2, *M. zeae* JJ-247; 3, *M. campisalis* SA2-6; 4, *M. stamsii* BoGlc83; 5, *M. thioparans* BMP-1; 6, *M. boroniphilus* T-15Z; 7, *M. subterraneus* COOI3B; and 8, *M. jeotgali* YKJ-10. *Peribacillus* clade is as follows: 1, *P. kribbensis* BT080; 2, *P. cavernae* L5*;* 3, *P. asahii* A001; 4, *P. psychrosaccharolyticus* 23296; 5, *P. muralis* LMG 20238; 6, *P. frigoritolerans* DSM 8801; 7, *P. implex* NBRC 15720; and *P. simplex* LMG 11160. *Bacillus* clade is as follows: 1, *B. aerophilus* 28K; 2, *B. stratosphericus* 1KF2a; 3, *B. licheniformis* DSM 13; 4, *B. licheniformis* ATCC 14580; 5, *B. velezensis* FZB42; 6, *B. vallismortis* DSM 11031; 7, *B. subtilis* IAM 12118; 8, *B. inaquosorum* BGSC 3A28; 9, *B. halotolerans* DSM 8802; and 10, *B. niacini* IFO15566

Blastn search of the entire 16S rRNA gene sequence of HI03-3b showed homology with those from the family Bacillaceae. Further phylogenetic analysis of the HI03-3b’s 16S rDNA sequence with those from some representative genera within Bacillaceae (*Bacillus*, *Cytobacillus*, *Mesobacillus*, *Neobacillus*, and *Peribacillus*) showed that HI03-3b was most closely related to *Cytobacillus* especially within the *C. firmus* clade (Fig. 2). Based on this outcome, we performed genome sequence multiple alignment between HI03-3b and three closely related species (*C. firmus* NCTC10335, *C. oceanisedirmins* YPW-V2, and *C. oceanisedirmins* 2691) using Mauve [13]. It was found some differences in genomic composition among them, as visualized in Fig. S3.

**Fig. 3:**
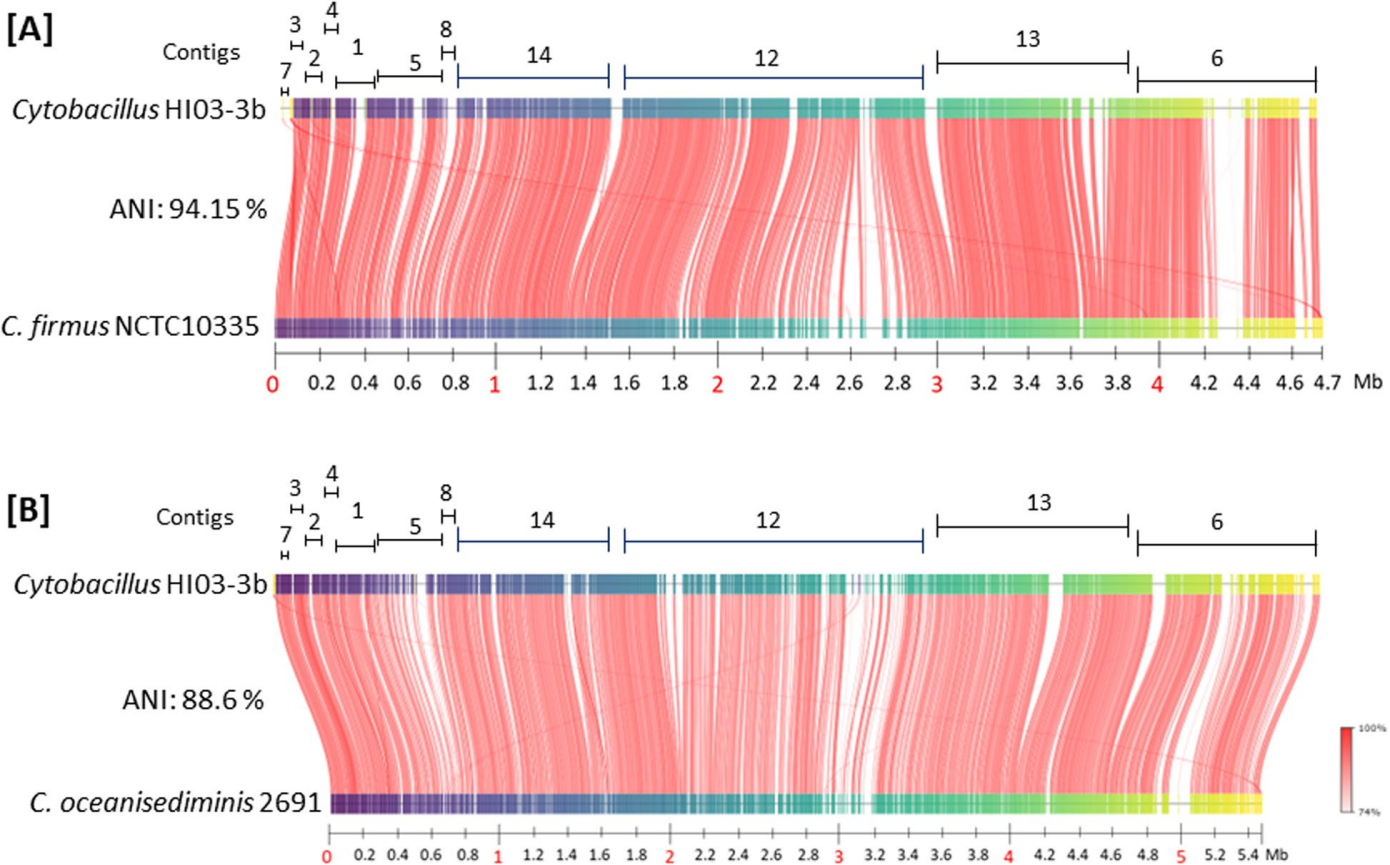
Pairwise genome sequence comparison to determine the order and direction of HI03-3b contigs. **A** HI03-3b contigs were mapped onto with the genome sequence of *C. firmus* NCTC10335 (Acc. Nu. NZ_UFTC01000001.1) with the ANI score of 94.15 %. **B** HI03-3b contigs were compared with the complete genome sequence of *C. oceanisediminis* 2691 (NCBI Acc. Nu. GCA_000294775.2) with the ANI score of 88.6 %

Furthermore, based on MiGA [52], HI03-3b genome sequence showed the most similar ANI (average nucleotide identity) score of 94.1 % and AAI (average amino acid identity) score of 95 % to those of *C. firmus* NCTC10335 (GenBank assembly accession: GCA900445365). The next top hits were *Sporosarcina globispora* DSM 4 [GenBank assembly accession: GCA001274725.1] and *C. oceanisediminis* CGMCC 1.10115 [GCA007830235] with the AAI/ANI scores of 88.1%/86.7 and 82.81%/81.46%, respectively. To determine the order and direction of the 11 contigs of HI03-3b genome sequence, we run Blastn [2] for pairwise DNA-DNA sequence comparison using the genome sequences of *C. oceanisedirmins* 2691 and *C. firmus* NCTC10335 as the subject sequences (Table S1). To validate the order and direction of HI03-3b contigs, we then carried out ANI analysis [29] on PROKSEE with CGView Server [58, 59] using *C. oceanisedirmins* 2691 and *C. firmus* NCTC10335 as the references, respectively (Fig. 3).

Finally, we run Genome-to-Genome Distance Calculator (GGDC) 3.0 [41] to confirm the similarity level of the genome sequences between HI03-3b and *C. firmus* NCTC10335 as the closest taxa. The GGDC analysis revealed a recommended a DNA-DNA hybridization (DDH) of 57.60 % to *C. firmus* NCTC10335. This value is lower than the new species threshold (DDH > 70.0% for the same species and DDH > 79.0% for the same subspecies). The difference in % G+C between the two genome sequences was 0.30. The genome comparative and phylogenetic studies suggest that HI03-3b is a novel species within *Cytobacillus*, and therefore, we propose the name as *Cytobacillus wakatobiense* HI03-3b (HI refers to Hoga Island, the place where it was derived from).

### Genome properties and primary metabolisms

**Fig. 4:**
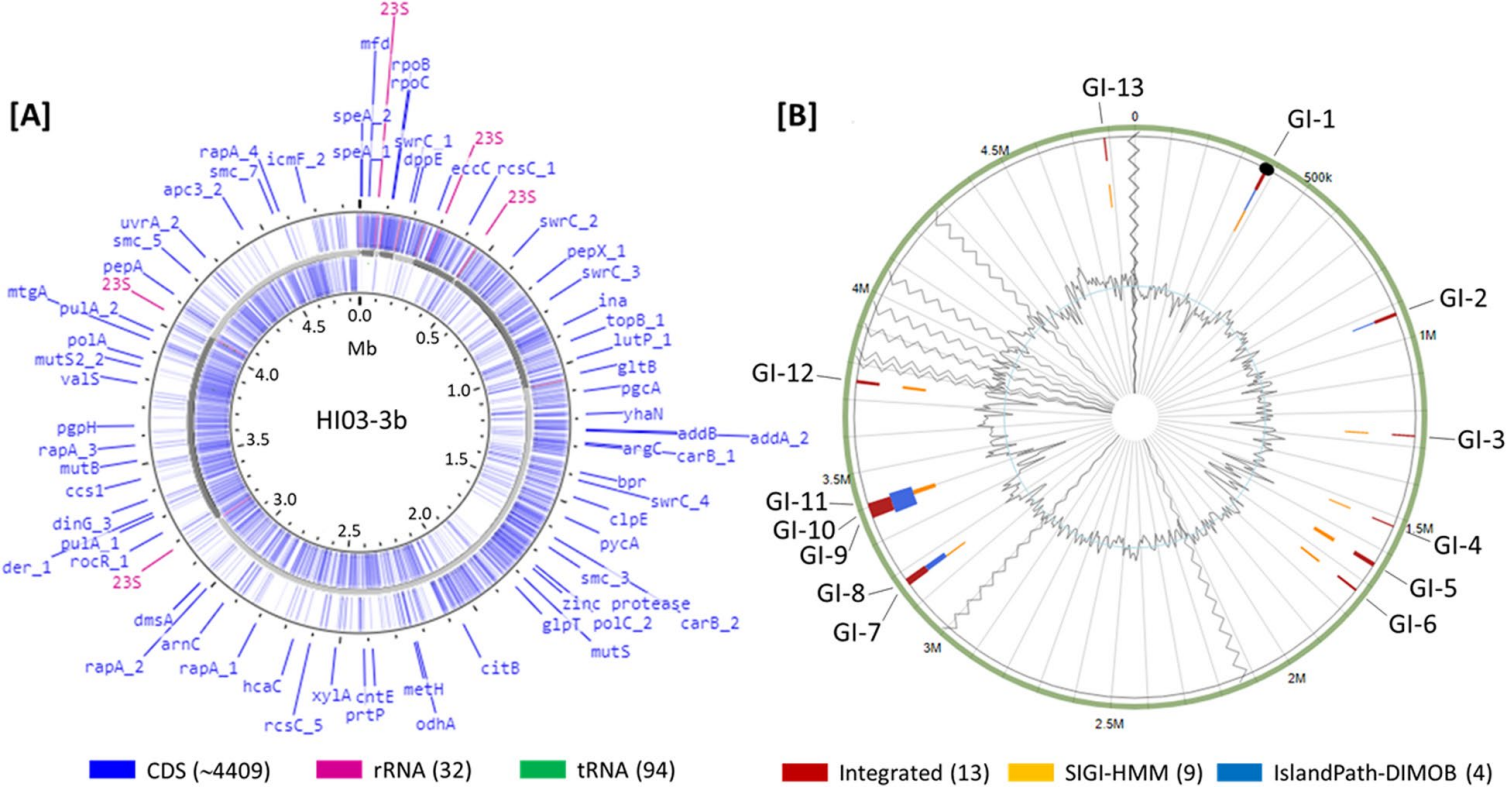
Visualization of HI03-3b genome features. **A** Identification of CDSs, rRNA genes, and tRNA genes (indicated by blue, purple, and green colours) using PROKSEE on the CGView Server [58, 59] with Prokka annotation [54]. The order and direction of contigs generated after genome sequence assembly were determined based on blastn pairwise alignment [2] using the genome sequences of *C. oceanisediminis* 2691 and *C. firmus* NCTC10335 as the references. **B** Prediction of genomic islands using IslandViewer 4 based on three different methods (indicated by red, orange, and blue colours) [5] with the genome sequence of *C. oceanisediminis* YPW-V2 as the reference. Note: CDS, coding sequence; GI, genomic island

By referring to Blastn comparison and ANI analysis in Fig. 3, the 11 contigs of the assembled HI03-3b genome sequence were visualized in the right order and direction using PROKSEE on the CGView Server [58, 59] with Prokka annotation [54]. The results showed HI03-3b genomic features, such as open reading frames (ORFs), CDSs (coding sequences), 94 genes for tRNAs, and 32 genes for rRNAs (5S, 16S, and 23S) (Fig. 4A). The sequenced HI03-3b genome harboured 5655 ORFs based on GeneMark.hmm PROKARYOTIC Version 3.26 analysis [6] and 4409 CDSs based on the NCBI record (Table S2). Furthermore, tRNAscan-SE 2.0 [9, 37]. showed 24 types of tRNAs encoded on HI03-3b genome, which were dominated by tRNAs for Gly, Glu, and Leu (Table S3). Based on KofamKOALA [3], 12 of 32 rRNA genes belong to 16S rRNA genes (Table S4). By aligning with the reference *C. oceanisediminis* YPW-V2 on IslandViewer 4 [5], we found 13 genomic islands in HI03-3b, including polysaccharide biosynthesis gene cluster, based on the integrated methods of SIGI-HMM [66] and IslandPath-DIMOB [28] (Fig. S4B). However, no pathogenic islands/genes were detected within the HI03-3b genome [4].

The sequences of all ORFs or total genes resulted from GeneMarkS analysis [6] were analysed further using KofamKOALA [3] against KOfam, a customized HMM database of KEGG Orthologs (KOs) to predict HI03-3b primary metabolisms. The results showed the presence of genes encoding enzymes involved in carbohydrate metabolisms, such as glycolysis, gluconeogenesis, citrate cycle, pentose phosphate pathway, Entner-Doudoroff pathway, and glycogen degradation [3] (Table S5 for the detail). Other metabolisms identified in this genome based on KofamKOALA [3] include carbon fixation, sulphur metabolism, fatty acid metabolism, purine metabolism, metabolisms of amino acids, shikimate pathway, lipopolysaccharide metabolism, polyamine biosynthesis, and cofactor and vitamin metabolism (Tables S6–S12 for the detail) [3].

### Genes encoding enzymes with antimicrobial potential

**Fig. 5:**
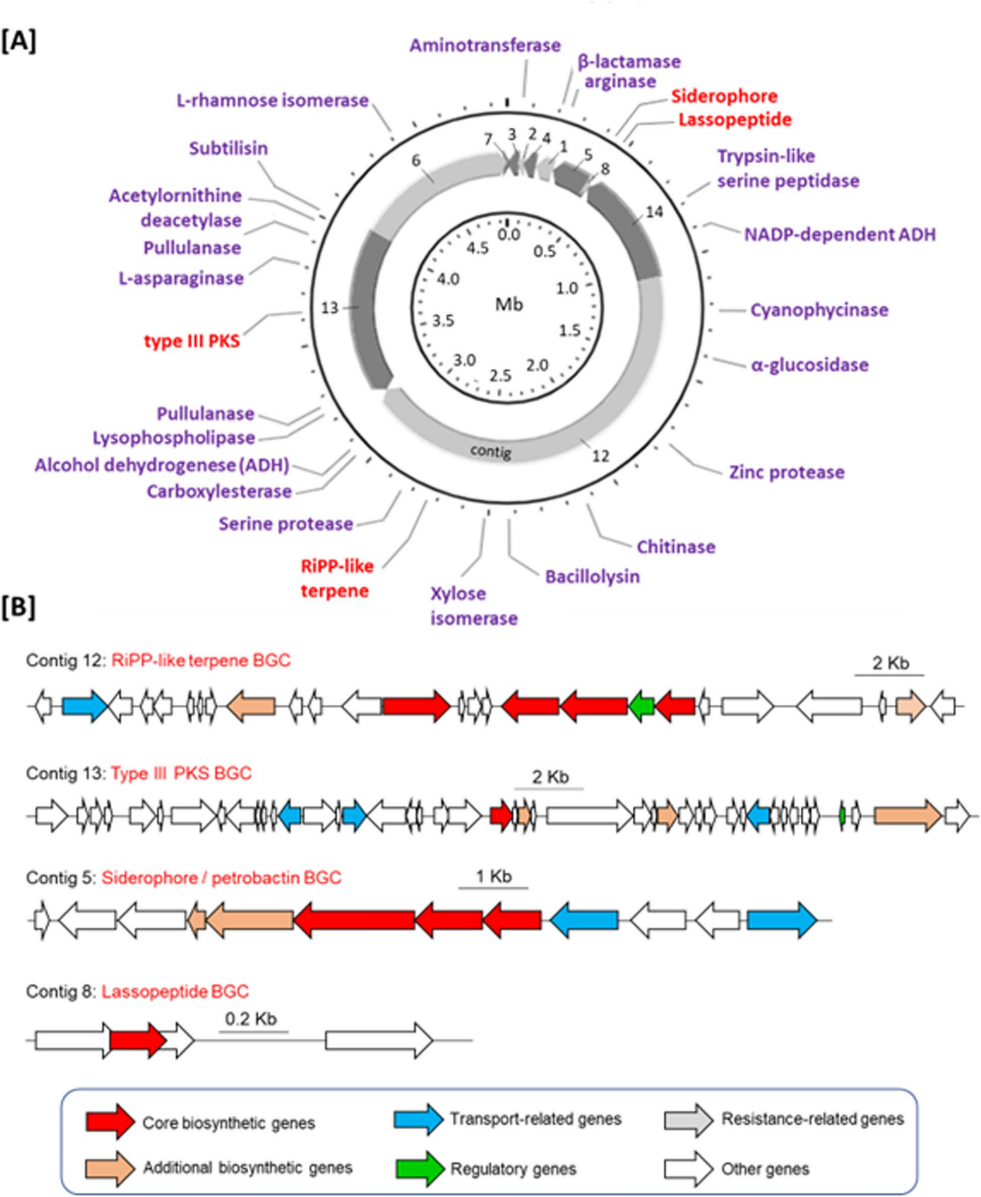
The position of biotechnologically potential genes on the HI03-3b genome map. **A** Certain loci encoding biotechnologically potential enzymes and secondary metabolite biosynthesis were determined based on PROKSEE analysis on the CGView Server [58, 59]. **B** Natural product BGCs were identified in the HI03-3b genome sequence based on antiSMASH analysis [7] supported by BLASTx [2], showing the presence of core and additional biosynthetic genes, transport-related genes, regulatory genes, and other genes. The core biosynthetic genes of RiPP-like terpene BGC (from left to right) are predicted to code for squalene-hopene cyclase, leader peptide (SagB-type dehydrogenase domain), YcaO cyclodehydratase, and thiazole-containing bacteriocin maturation protein. PKS type III BGC contains a chalcone synthase gene (indicated by red colour) as the core biosynthetic gene. The siderophore BGC harbours the core biosynthetic genes encoding lucA/lucC family proteins. Based on RiPPMiner prediction [1] the core lassopeptide BGC encodes a precursor peptide that consists of a leader (VKAPGSTGEGHWKLGNLSAEEKSGIPRVAVKCVEMWRNTSGE) and core peptide sequence (GDSLVCN). A crosslink may occur between serine (S) and cysteine (C) residues in the core peptide [1]. Note: PKS, polyketide synthase; BGC, biosynthetic gene cluster; RiPP, ribosomally synthesized and post-translationally modified peptides

Based on the carbohydrate-active enzyme (CAZy) database [8], it was found that the HI03-3B genome harbours some genes encoding carbohydrate active enzymes (Table S13), including glycoside hydrolases (GHs) and glycosyltransferases (GTs). Some of these genes encoding enzymes that might be related to the degradation of polysaccharides, such as putative type 1 pullulanase (GH13 family), chitinase (GH18 family), and α-glucosidase (GH65 family) based on KEGG Orthology search [3] (Table 1). KEGG Orthology search [3] combined with BLASTx analysis [2] also showed the presence of other genes encoding biotechnologically potential enzymes, such as arginase, cyanophycinase, protease, bacillolysin, xylose isomerase, and alcohol dehydrogenase [35] (Table 1 and see also Table S14 with notes describing their potential applications). Using PROKSEE on the CGView Server [58, 59], we annotated some biotechnologically potential genes on the circular HI03-3b genome map by referring to their position on contigs from the outcome of GeneMarkS analysis [6] (Fig. 5A).

**Table 1:** Some biotechnologically promising genes in HI03-3b, which encode putative enzymes with potential anti-biofilm activities

Gene ID	AA	KEGG Orthology search	BLASTx search		Proposed function
			Protein & acc. no.	I/S (%)	
HI03-309	214	Cyanophycinase [EC:3.4.15.6]	Cyanophycinase [SUV02533.1]	89/95	Cyanophycinase
HI03-932	411	Zinc protease [EC:3.4.24.-]	Insulinase family protein [WP_227887042.1]	99/100	Zinc-dep. metalloproteinase
HI03-1104	1395	Minor extracellular serine protease Vpr [EC:3.4.21.-]	S8 family serine peptidase [WP_197246214.1]	100/100	Serine peptidase
HI03-1355	387	Chitinase [EC:3.2.1.14]	Glycosyl hydrolase family 18 [WP_227887042.1]	98/99	Chitinase [WP_248347211.1]
HI03-1694	593	Bacillolysin [EC:3.4.24.28]	M4 metallopeptidase [WP_222500569.1]	99/99	Bacillolysin [WP_248347527.1]
HI03-2166	449	Serine protease aprx. [EC:3.4.21.-]	S8 family peptidase [WP_226617693.1]	99/99	Serine protease [WP_248347905.1]
HI03-2670	521	Pullulanase [EC:3.2.1.41]	Type 1 pullulanase [WP_222500182.1]	99/99	Pullulanase
HI03-3302	306	L-Asparaginase [EC:3.5.1.1]	Asparaginase [WP_222497870.1]	100/100	L-Asparaginase [WP_248348545.1]
HI03-3438	958	Pullulanase [EC:3.2.1.41]	Type 1 pullulanase [WP_222500182.1]	99/99	Pullulanase [WP_248348709.1]
HI03-3561	391	Leader peptide-processing serine protease [EC:3.4.21.-]	S8 family serine peptidase [WP_048011423.1]	99/99	Subtilisin [WP_248348651.1]

*Abbreviations: AA* amino acid size, *I* identity, *S* similarity

### Biosynthetic genes of secondary metabolites

We also found putative genes involved in secondary metabolite biosynthesis (Fig. 5), such as those coding for enzymes responsible for terpenoid backbone biosynthesis predicted using KEGG Orthology [3]. Further analysis using AntiSMASH version 6.0 [7] allowed us to identify the presence of a biosynthetic gene cluster (BGC) of ribosomally synthesized and post-translationally modified peptides (RiPP)-like terpene in the contig 12 of HI03-3b genome (Fig. 5, Fig. S5 for the detail), which was verified by Bagel4 analysis [15]. Other BGCs identified in this sequenced genome code for polyketide ketosynthase (PKS) type 3 in the contig 13 (Fig. 5, Fig. S6 for the detail), siderophore/petrobactin BGC (Fig. 5, Fig. S7), and a lassopeptide BGC in the contig 8 (Fig. S8). Further research is required to isolate secondary metabolites and enzymes from this HI03-3b strain in order to test them against biofilm-forming pathogens.

### Genome sequence data and strain availability

We deposited our HI03-3b genome sequence in the GenBank database, which was publicly available under the accession number JAKDDU000000000.1 (BioProject ID: PRJNA785558, and BioSample: SAMN23566444). Some of the biotechnologically potential genes in HI03-3b have been annotated by GenBank with the following accession numbers: WP_248347211.1 (chitinase/glycosyl hydrolase family 18 protein), WP_248347527.1 (bacillolysin/M4 family metallopeptidase), WP_248347905.1 (serine protease/S8 family peptidase), WP_248348545.1 (L-asparaginase), WP_248348709.1 (pullulanase), WP_248348651.1 (subtilisin/S8 family serine peptidase), WP_248349705.1 (L-rhamnose isomerase), and WP_248349198.1 (nucleoside hydrolase). This HI03-3b strain was registered in National Center for Biotechnology Information (NCBI) with the taxonomy ID 2862822. Authors maintain this HI03-3b strain at the Microbiology Laboratory of Universitas Muhammadiyah Semarang and at National Research and Innovation Agency (BRIN), Indonesia.

## Discussion

We had isolated a biotechnologically potential bacterial epibiont, designated as HI03-3b, from the surface of the Indonesian brown algae *Hydroclathrus* sp. The ability of this epibiont to degrade complex polysaccharide and to inhibit a pathogenic gram-positive bacterium had motivated us to sequence the whole genome. HI03-3b genome sequence showed the highest AAI and ANI scores to *C. firmus* NCTC10335, which was supported by 16S rRNA gene phylogenetic analysis. Further genome comparison between HI03-3b and three closely related *Cytobacillus* species, including NCTC10335 strain, suggested some differences in genomic composition among them. Further GGDC analysis indicated that a recommended DNA-DNA hybridization (DDH) between the two strains is lower than the new species threshold (DDH > 70.0% for the same species). The genome features, such as the numbers of protein-coding genes (CDSs), tRNAs, and rRNAs, were different between HI03-3b and *C. firmus* NCTC10335 (Table S2). Taken all together, we propose HI03-3b as a novel species within *Cytobacillus* genus, and therefore, we named it *Cytobacillus wakatobiense* HI03-3b.

The genome sequence analysis provided insights into metabolic pathways, which helped us to understand the ecological role and biotechnological importance of HI03-3b. For example, some genes identified in this HI03-3b genome are involved in the biosynthesis of vitamins, such as thiamine (B1), riboflavin (B2), pantothenate (B5), pyridoxine (B6), biotin (B7), lipoic acid, cobalamin (B12), and menaquinone (K2) (see Table S11). This indicates that HI03-3b may play a crucial ecological role in providing the algal host with vitamins for growth and development [16]. From biotechnological perspective, the presence of these vitamin biosynthetic pathways suggests that HI03-3b could be harnessed as a natural alternative for industrial vitamin production.

We also found the presence of genes involved in polyamine biosynthesis in HI03-3b, which leads to spermidine formation (see Fig. S4 for spermidine biosynthetic pathway). The human gut bacteria *Bacteroides thetaiotaomicron* and *Fusobacterium varium* represent other bacterial species reported to synthesize spermidine both in vivo and in vitro [49]. From medical perspective, spermidine is known as a bioactive metabolite that can extend life span in model organisms, suggesting its potential application in delaying ageing and promoting longevity in human [39]. Spermidine has particularly been shown to increase epithelial renewal and anti-inflammatory macrophage development in the colon, highlighting its importance in the maintenance of intestinal homoeostasis and immunity [46]. From ecological point of view, spermidine was found essential for robust biofilm formation in *Bacillus subtilis* [25]. In addition to spermidine, TatD DNase can contribute to biofilm formation, as has recently shown in the biofilm formation of the bacterium *Trueperella pyogenes* [69]*.* Interestingly, TatD DNase-encoding gene was found in HI03-3b genome. Taken together, we propose that HI03-b may rely on molecules such as spermidine and TatD DNase to promote its colonization and biofilm formation on the macroalgal surface.

Among 4409 protein-coding sequences identified in this strain, some genes encode enzymes for polysaccharide and protein degradation known to exhibit anti-biofilm properties against pathogenic bacteria [57]. Notable examples are protease [50], chitinase [12], a mixture of bacillolysin and subtilisin (Protamex®) [20, 48], L-asparaginase [64, 65], and pullulanase [51] (Table 1). From the ecological point of view, the presence of these genes suggests that HI03-3b may produce antimicrobial or anti-biofilm enzymes in competing for nutrient and space against other epibionts as well as in protecting algal host from predation [17, 18]. This was supported by our finding that the extracellular cell-free supernatant of HI03-3b exhibited antimicrobial activity against the bacterial pathogen *S. aureus*.

Other biotechnologically potential genes identified in this HI03-3b include those encoding arginase, xylose isomerase, carboxylesterases, phospholipases, and arginine decarboxylase (see Table S14). Arginase for example has been used for the environmentally friendly preparation of L-ornithine as food supplement and nutrition product [34]. Xylose isomerase has widely been used in the production of high-fructose corn syrup (HFCS) and bioethanol [47]. It is potentially explored to produce some value-added chemicals in the food, cosmetics, and pharmaceutical industries [44]. Carboxylesterases (CEs) have been applied in xenobiotic and endobiotic degradations, biocatalysis, and drug metabolism [68]. Phospholipases have been used in scientific and medical research, such as inhibitors for generating anti-inflammatory agents and as diagnostic markers for microbial infections [30, 61]. Arginine decarboxylase catalyses conversion of L-arginine into agmatine, a valuable pharmaceutical intermediate with various potential therapeutic functions in neurotransmitter systems, nitric oxide synthesis, and polyamine metabolism [60].

HI03-3b genome harbors a RiPP-like terpene BGC predicted to encode the biosynthesis of thiazole-containing heterocyclic bacteriocin as a new subfamily of ribosomally synthesized peptides antimicrobial peptides (AMPs) ([23, 33]). This RiPP-like terpene BGC is characterized by the presence of genes encoding proteins involved in heterocycloanthracin biosynthesis, such as toxin precursor, SagB-type dehydrogenase domain (nitroreductase family), serine protease, YcaO cyclodehydratase, thiazole-containing bacteriocin maturation protein, and a transport protein [23] (Fig. S5). Bacteriocins that belong to heterocycloanthracin family are known as antimicrobial and antibiofilm agents [53], best exemplified by sonorensin that exhibited antibiofilm activity against *S. aureus* and food bio-preservative potential [10, 11]. It was reported that bacteriocins of heterocycloanthracin family are initially synthesized as biologically inactive peptides (protoxins or precursors) with an N-terminal leader peptide. This protoxin subsequently undergoes enzymatic modifications that involve the cleavage of the leader peptide by protease and the formation of a thiazole or oxazole ring by cyclodehydratase and dehydrogenase. The resulting mature toxin is then exported by a transport protein [23, 33]. Validation of the function of this RiPP-like terpene BGC in HI03-3b through gene mutagenesis or heterologous expression and subsequent identification of the bacteriocin produced are necessary to explore the biotechnological potential as an antibiofilm and food preservative agent.

We compared the presence of some biotechnologically potential genes between *Cytobacillus* HI03-3b and the most closely related strain *C. firmus* NCTC10335 (Table S15). It was found that several genes encoding carbohydrate active enzymes in HI03-3b, such as β-glucosylceramidase [EC:3.2.1.45], xylose isomerase [EC:5.3.1.5], L-rhamnose isomerase [EC:5.3.1.14], chitinase [EC:3.2.1.14], and arginase [EC:3.5.3.1], were absent in NCTC10335. In contrast, α-amylase [EC:3.2.1.1] identified in NCTC10335 was not found in HI03-3b. Another difference is that HI03-3b contained lassopeptide BGC, while NCTC10335 harboured lanthipeptide class 2. Some potential genes present in both strains occur in different copy numbers (Table S15), such as those encoding oligo-1,6-glucosidase [EC:3.2.1.10], α-glucosidase [EC:3.2.1.20], zinc protease [EC:3.4.24.-], serine protease [EC:3.4.21.-], bacillolysin [EC:3.4.24.28], beta-lactamase class A [EC:3.5.2.6], and acetylornithine deacetylase [EC:3.5.1.16]. The difference in genome features and gene composition between *Cytobacillus* HI03-3b and its closely related strain strongly suggests the uniqueness of HI03-3b that would enable it to adapt to the algal surface environment. This was supported by the finding of 13 genomic islands in HI03-3b that possibly occur through horizontal gene transfer, which may provide this epibiont with adaptive traits to live and survive on the surface of brown algae [4].

## Conclusion

The whole-genome sequence analysis of the brown algae-associated epibiont *Cytobacillus* sp. HI03-3b showed the presence of biotechnologically potential genes, including those encoding commercially useful enzymes, such as chitinase, pullulanase, protease, bacillolysin, subtilisin, and L-asparaginase, as well as biosynthetic genes for secondary metabolites and vitamins. These might ecologically be important as strategies of the epibiont to outcompete with other bacteria during biofilm formation, protect its algal host from pathogenic infection, and provide the host with necessary vitamins. Some of such metabolic genes could potentially be exploited as a basis for the development of anti-infective agents against pathogenic biofilms. Further functional studies by gene heterologous expression, and mutagenesis or enzyme activity assays, are necessary to validate the function of these genes.

## Supplementary Information

**Additional file 1: Figure S1.** Visualization and quality assessment of whole H103-3b genome assembly. [A] Workflow of HI03-3b genome sequence assembly. [B] Quality parameters of the assembled HI03-3b genome sequence using Quast [22]. [C] GC content of the assembled HI03-3b genome sequence against density of reads using RSeQC [67]. **Figure S2.** Taxonomic distribution analysis based on MyTaxa Scan result from MiGA ([38, 52]) on the assembled HI03-3b genome sequence, showing that the high quality genome (the major light blue) with minor contamination. **Figure S3.** Genome comparison of HI03-3b with three closely related *Cytobacillus* strains using Mauve [13]. Each colored block represents a region of the HI03-3b genome sequence, which align with parts of other genomes. Blocks above the central line show forward orientation relative to the first genome sequence, while blocks below the central line indicate regions aligning in the reverse complement orientation. Inside each block, the height of the similarity profile correlates with the average level of conservation region of the genome. **Figure S4.** Genes involved in spermidine biosynthesis identified in the HI03-3b genome, which were predicted based on KofamKOALA [3] and BLASTx [2]. Notes: spermidine has been shown to increase epithelial renewal and anti-inflammatory macrophage development in the colon, highlighting its importance in the maintenance of intestinal homoeostasis and immunity [46]. From medical perspective, spermidine has been known to extend life span in model organisms, indicating its potential application in delaying aging and promoting longevity in human [39]. **Figure S5.** RiPP-like terpene BGC identified in the HI03-3b genome, which were predicted based on antiSMASH [7] and BLASTx [2]. **Figure S6.** PKS type III BGC identified in the HI03-3b genome, which were predicted based on antiSMASH [7] and BLASTx [2]. **Figure S7.** Siderophore/petrobactin BGC and lassopeptide BGC identified in the HI03-3b genome, which were predicted based on antiSMASH [7] and BLASTx [2]. **Table S1.** The order and direction of HI03-3b contigs were validated by comparing with the genome sequences of *C. oceanisedirmins* 2691 and *C. firmus* NCTC10335. **Table S2.** Comparation of genomic features between *Cytobacillus* HI03-3b (in this work) and *C. firmus* NCTC10335 (GenBank assembly accession number GCA_900445365.1). **Table S3.** Estimated numbers of tRNAs in HI03-3b genome sequence analyzed using tRNAscan-SE 2.0. ([9] [37];). **Table S4.** Numbers of 16S-rRNAs in HI03-3b genome sequence based on KofamKOALA [3]. **Table S5.** Predicted carbohydrate metabolisms in HI03-3b based on KofamKOALA [3]. **Table S6.** Energy metabolisms in HI03-3b based on KofamKOALA [3] analysis. **Table S7.** Lipid metabolisms in HI03-3b based on KofamKOALA [3] analysis. **Table S8.** Nucleotide metabolisms in HI03-3b based on KofamKOALA [3] analysis. **Table S9.** Predicted amino acid metabolisms in HI03-3b based on KofamKOALA [3]. **Table S10.** Predicted glycan metabolism in HI03-3b based on KofamKOALA [3]. **Table S11.** Carbon and vitamins metabolisms in HI03-3b based on KofamKOALA [3]. **Table S12.** Predicted terpenoid biosynthesis in HI03-3b based on KofamKOALA [3]. **Table S13.** Some genes encoding carbohydrate active enzymes. **Table S14.** Genes on HI03-3b genome encoding biotechnologically potential enzymes. **Table S15.** Comparation of biosynthetically potential genes between *Cytobacillus* HI03-3b and *C. firmus* NCTC10335.

## Data Availability

We deposited our HI03-3b genome sequence in the GenBank database, which was publicly available under the accession number JAKDDU000000000.1 (BioProject ID: PRJNA785558 and BioSample: SAMN23566444). Some of the biotechnologically potential genes in HI03-3b have been annotated by GenBank with the following accession numbers: WP_248347211.1 (chitinase/glycosyl hydrolase family 18 protein), WP_248347527.1 (bacillolysin/M4 family metallopeptidase), WP_248347905.1 (serine protease/S8 family peptidase), WP_248348545.1 (L-asparaginase), WP_248348709.1 (pullulanase), and WP_248348651.1 (subtilisin/S8 family serine peptidase). This HI03-3b strain was registered in National Center for Biotechnology Information (NCBI) with the taxonomy ID 2862822. Authors maintain this HI03-3b strain at the Microbiology Laboratory of Universitas Muhammadiyah Semarang and at National Research and Innovation Agency (BRIN), Indonesia.

## Abbreviations

ORF: Open reading frame
CDS: Coding sequence
MS: Minimal seawater
ANI: Average nucleotide identity
AAI: Average amino acid identity
CAZy: Carbohydrate-active enzyme
DDH: DNA-DNA hybridization
BGC: Biosynthetic gene cluster
NP: Natural products
