# Supplementary material for: Biotechnologically potential genes in a polysaccharide-degrading epibiont of the Indonesian brown algae Hydroclathrus sp
Source: J Genet Eng Biotechnol. 2023 Feb 14;21:18. doi: 10.1186/s43141-023-00461-5 (PMC9928984; doi:10.1186/s43141-023-00461-5)
Supplement: Supplementary file 1 — Additional file 1: Figure S1. Visualization and quality assessment of whole H103-3b genome assembly. [A] Workflow of HI03-3b genome sequence assembly. [B] Quality parameters of the assembled HI03-3b genome sequence using Quast [22]. [C] GC content of the assembled HI03-3b genome sequence against density of reads using RSeQC [67]. Figure S2. Taxonomic distribution analysis based on MyTaxa Scan result from MiGA ([38, 52]) on the assembled HI03-3b genome sequence, showing that the high quality genome (the major light blue) with minor contamination. Figure S3. Genome comparison of HI03-3b with three closely related Cytobacillus strains using Mauve [13]. Each colored block represents a region of the HI03-3b genome sequence, which align with parts of other genomes. Blocks above the central line show forward orientation relative to the first genome sequence, while blocks below the central line indicate regions aligning in the reverse complement orientation. Inside each block, the height of the similarity profile correlates with the average level of conservation region of the genome. Figure S4. Genes involved in spermidine biosynthesis identified in the HI03-3b genome, which were predicted based on KofamKOALA [3] and BLASTx [2]. Notes: spermidine has been shown to increase epithelial renewal and anti-inflammatory macrophage development in the colon, highlighting its importance in the maintenance of intestinal homoeostasis and immunity [46]. From medical perspective, spermidine has been known to extend life span in model organisms, indicating its potential application in delaying aging and promoting longevity in human [39]. Figure S5. RiPP-like terpene BGC identified in the HI03-3b genome, which were predicted based on antiSMASH [7] and BLASTx [2]. Figure S6. PKS type III BGC identified in the HI03-3b genome, which were predicted based on antiSMASH [7] and BLASTx [2]. Figure S7. Siderophore/petrobactin BGC and lassopeptide BGC identified in the HI03-3b genome, which were pre [file 43141_2023_461_MOESM1_ESM.docx]

**Supplementary Material**

**Biotechnologically potential genes in a polysaccharide-degrading epibiont of the Indonesian brown algae *Hydroclathrus* sp.**

Stalis Norma Ethica^1*^, Dewi Seswita Zilda^2*^, Oedjijono Oedjijono^3^, Muhtadi Muhtadi^4^, Gintung Patantis^2^, Sri Darmawati^1^, Sri Sinto Dewi^5^, Agus Sabdono^6^, Agustinus Robert Uria^7ζ^

^1^ Magister Program of Clinical Laboratory Science, Universitas Muhammadiyah Semarang (UMS)

Jalan Kedungmundu Raya, Semarang 50273, Indonesia. Email: norma@unimus.ac.id
^2^ Research and Development Center for Marine and Fisheries Product Processing and Biotechnology

Jalan KS Tubun Petamburan VI, Slipi, Jakarta Pusat 10260, Indonesia.

Email: seswitazilda@gmail.com, gintungpatantis@gmail.com

^3^ Faculty of Biology, Universitas Jenderal Soedirman, Purwokerto 53122, Indonesia. Email: oedjijono@hotmail.com

^4^ Faculty of Pharmacy, Universitas Muhammadiyah Surakarta, Sukoharjo 57162, Indonesia. Email: muhtadi@ums.ac.id

^5^ Diploma Study Program of Medical Laboratory Technology, Faculty of Nursing and Health Sciences,

Universitas Muhammadiyah Semarang, Semarang 50273, Indonesia. Email: sintomun@yahoo.com

^6^ Department of Marine Science, Faculty of Fisheries and Marine Science, Diponegoro University

Semarang 50272, Indonesia. Email: agus_sabdono@yahoo.com

^7^ Faculty of Pharmaceutical Sciences, Hokkaido University

Kita 12 Nishi 6 Kita-ku, Sapporo 060-0812, Japan. Email: auria@pharm.hokudai.ac.jp.

*Contributed equally

**^ζ^** corresponding authors


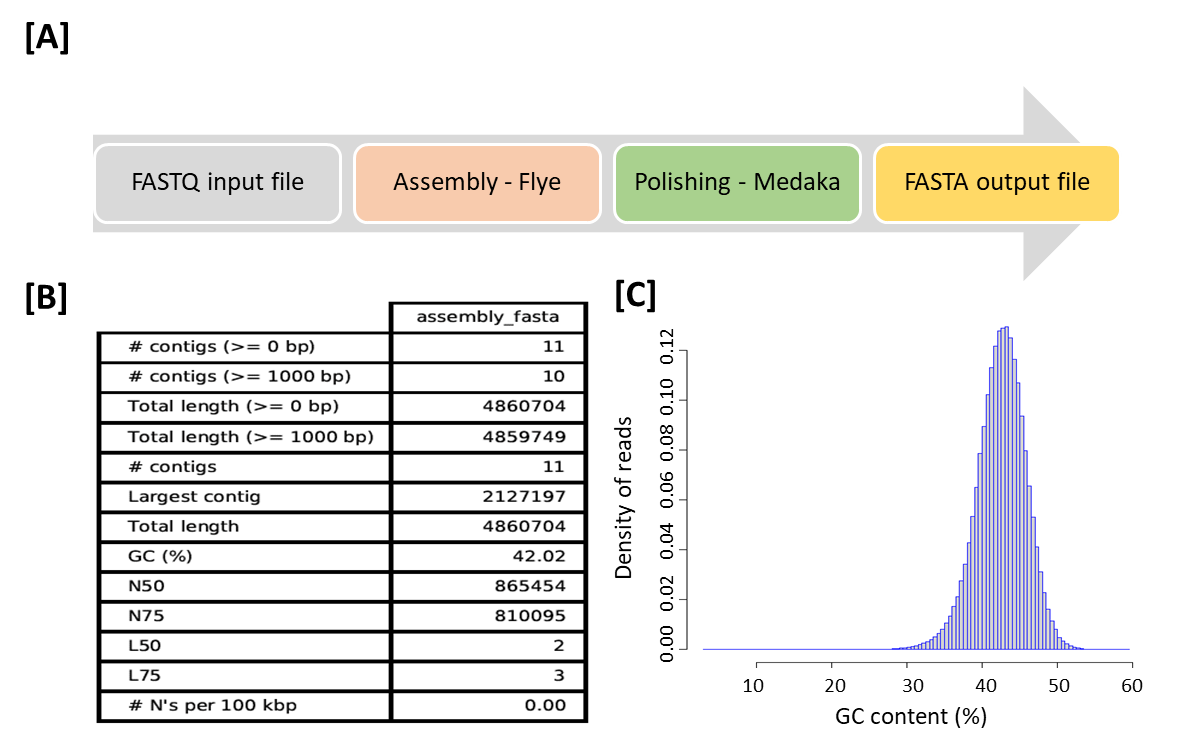


**Figure S1.** Visualization and quality assessment of whole H103-3b genome assembly. **[A]** Workflow of HI03-3b genome sequence assembly. **[B]** Quality parameters of the assembled HI03-3b genome sequence using Quast (Gurevich et al., 2013). **[C]** GC content of the assembled HI03-3b genome sequence against density of reads using RSeQC (Wang et al., 2012).


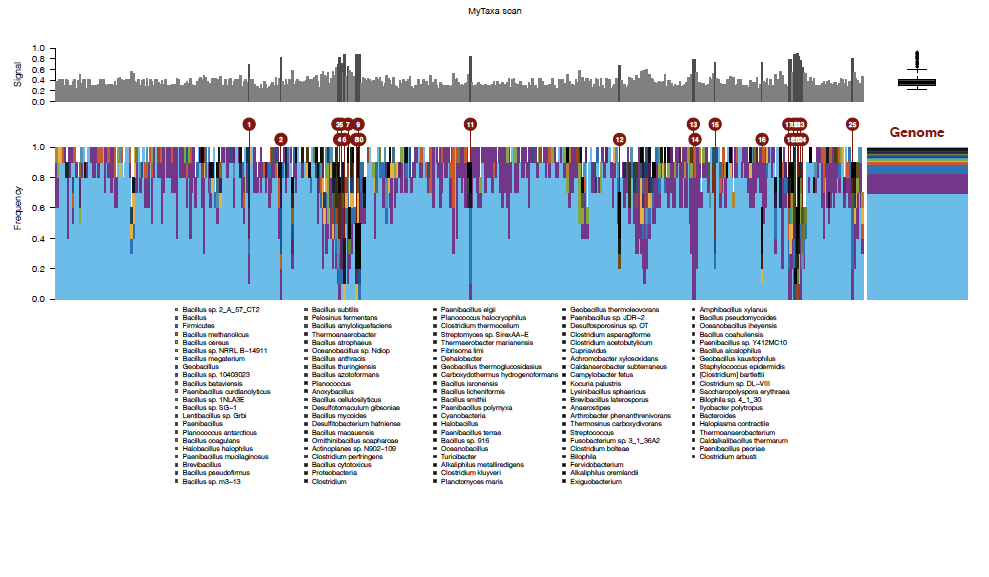


**
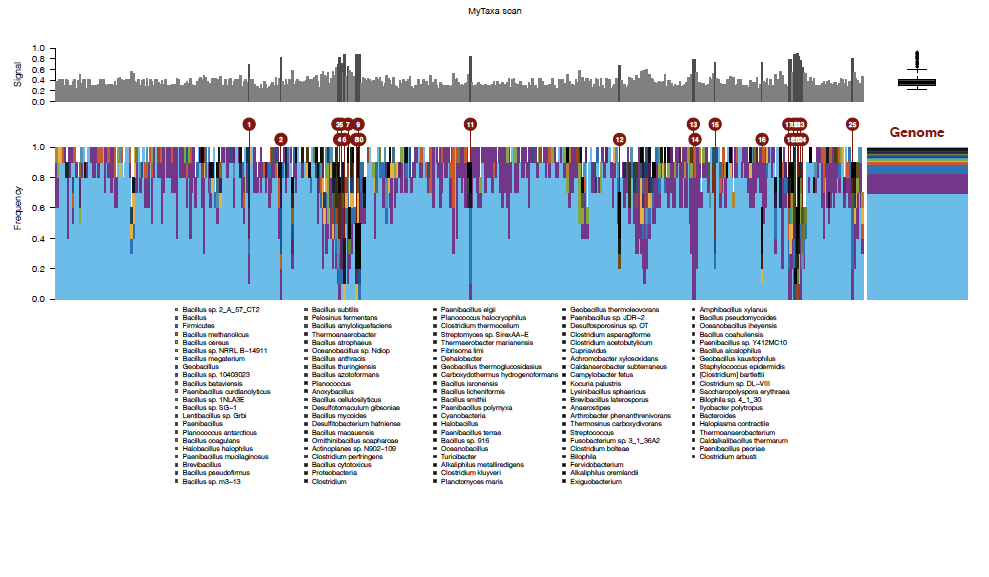
**

**Figure S2.** Taxonomic distribution analysis based on MyTaxa Scan result from MiGA (Luo et al., 2014; Rodriguez-R et al., 2018) on the assembled HI03-3b genome sequence, showing that the high quality genome (the major light blue) with minor contamination.


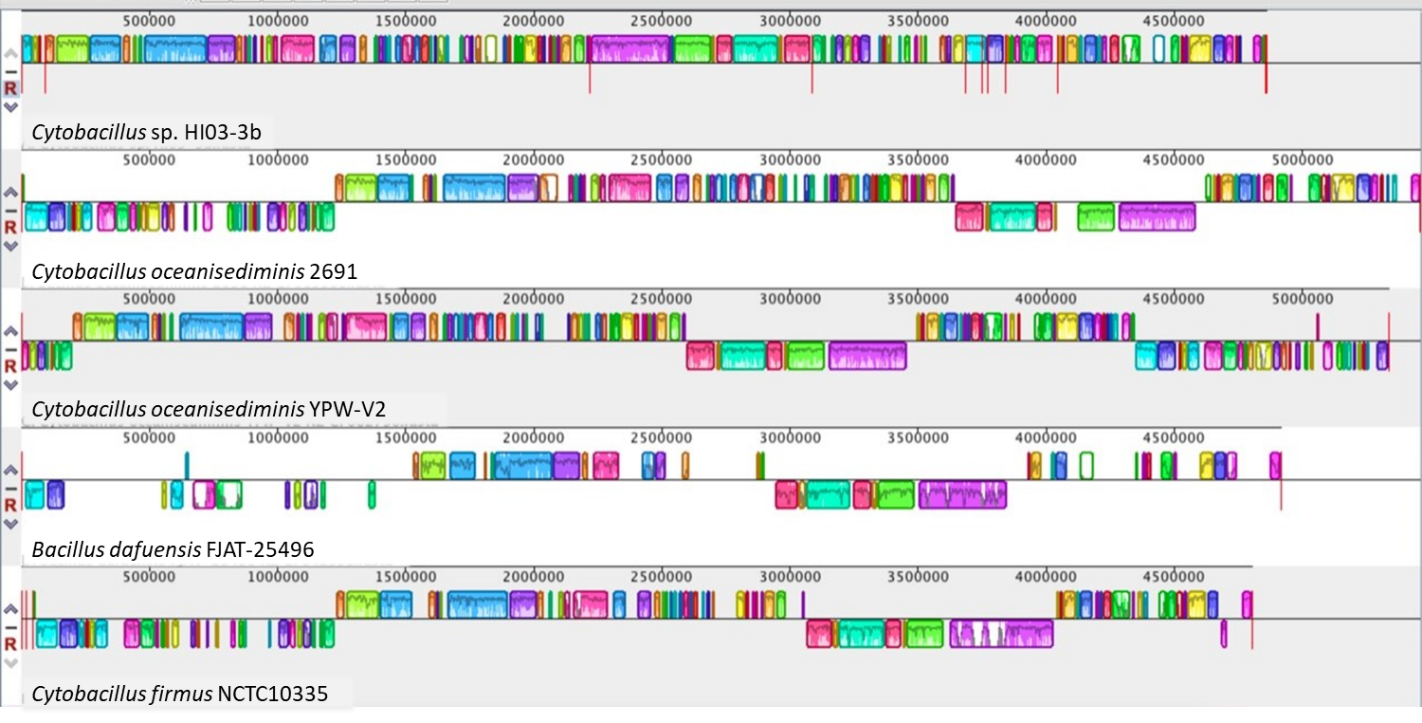


**Figure S3.** Genome comparison of HI03-3b with three closely related *Cytobacillus* strains using Mauve (Darling et al., 2004). Each colored block represents a region of the HI03-3b genome sequence, which align with parts of other genomes. Blocks above the central line show forward orientation relative to the first genome sequence, while blocks below the central line indicate regions aligning in the reverse complement orientation. Inside each block, the height of the similarity profile correlates with the average level of conservation region of the genome.

**Table S1**. The order and direction of HI03-3b contigs were validated by comparing with the genome sequences of *C. oceanisedirmins* 2691 and *C. firmus* NCTC10335

| **HI03-3b** | ***C. firmus* NCTC10335 [NZ_UFTC01000001.1]** | | | | | ***C. oceanisediminis* 2691 (GCA_000294775.2)** | | | | |
| --- | --- | --- | --- | --- | --- | --- | --- | --- | --- | --- |
| **Contig & size** | **relative position (bp)** | | **I (%)** | **C (%)** | **St.** | **relative position (bp)** | | **I (%)** | **C (%)** | **St.** |
| 7 (959 bp) | 12,840 | 13,424 | 99.81 | 94 | +/+ | 12866 | 13398 | 99.81 | 94 | +/+ |
| 3 (20951 bp) | 15,144 | 35,936 | 95.97 | 99 | +/- | 18,940 | 35,374 | 92.33 | 99 | +/- |
| 2 (63377 bp) | 35,560 | 89,674 | 91.07 | 99 | +/- | 35,560 | 113,348 | 91.98 | 99 | +/- |
| 4 (68696 bp) | 104,984 | 162,081 | 95.47 | 99 | +/- | 106,220 | 165,027 | 96.07 | 99 | +/- |
| 1 (92553 bp) | 173,179 | 288,027 | 97.12 | 96 | +/- | 173462 | 284650 | 88.37 | 98 | +/- |
| 5 (207137 bp) | 370,139 | 530,545 | 93.87 | 89 | +/- | 358248 | 341870 | 93.80 | 93 | +/- |
| 8 (2909 bp) | 392309 | 394107 | 98.67 | 98 | +/+ | 172639 | 175547 | 98.76 | 100 | +/+ |
| 14 (601380 bp) | 614,475 | 1,179,003 | 93.52 | 76 | +/- | 918615 | 931246 | 89.99 | 85 | +/- |
| 12 (2127197 bp) | 1,365,935 | 2,282,052 | 93.63 | 73 | +/+ | 1361801 | 2906270 | 88.27 | 86 | +/+ |
| 13 (865454 bp) | 3,062,058 | 3,691,424 | 96.19 | 96 | +/- | 3,697,504 | 4,624,004 | 89.14 | 97 | +/- |
| 6 (810095 bp) | 3,968,023 | 4,679,433 | 96.90 | 65 | +/+ | 4838455 | 4854294 | 91.13 | 76 | +/+ |

**Table S2** Comparation of genomic features between *Cytobacillus* HI03-3b (in this work) and *C. firmus* NCTC10335 (GenBank assembly accession number GCA_900445365.1)

| Properties | *Cytobacillus* HI03-3b  [GenBank GCA_023197125.1]  (In this work) | *C. firmus* NCTC10335  [GenBank GCA_900445365.1] |
| --- | --- | --- |
| Genome size | 4,860,704 bp | 4,803,910 |
| Open reading frames (ORFs) or total genes ^a^ | 5655 | 5307 |
| Coding genes or CDSs (with protein) ^b^ | 4409 | 4575 |
| tRNA genes ^b, c^ | 94 | 107 |
| rRNAs ^b^ | 32 | 36 |
| 23S-rRNAs ^b, d^ | 11 | 12 |
| 16S-rRNAs ^b, d^ | 11 | 12 |
| 5S-rRNAs ^b, d^ | 10 | 12 |
| ncRNAs ^b^ | 6 | 6 |

^a^) determined using GeneMark.hmm PROKARYOTIC (Version 3.26) (Besemer et al., 2001)

^b^) based on NCBI record

^c^) predicted with tRNAscan-SE 2.0. (Chan et al., 2021; Lowe and Chan, 2016)

^d^) estimated based on KofamKOALA (Aramaki et al., 2020)

**Table S3** Estimated numbers of tRNAs in HI03-3b genome sequence analyzed using tRNAscan-SE 2.0. (Chan et al., 2021; Lowe and Chan, 2016)

| Isotype/ | Contig | | | | | | | | | | | Total |
| --- | --- | --- | --- | --- | --- | --- | --- | --- | --- | --- | --- | --- |
| Anticodon | 1 | 6 | 5 | 4 | 3 | 2 | 14 | 13 | 7 | 8 | 12 |  |
| Ala | 1 | 1 |  |  |  |  | 1 | 1 |  |  |  | 4 |
| Arg | 1 | 1 |  |  |  |  | 1 | 2 |  |  | 1 | 6 |
| Asn | 2 |  |  |  |  |  |  | 1 |  |  | 1 | 3 |
| Asp | 1 | 1 | 1 |  |  |  | 1 | 1 |  |  | 1 | 6 |
| Cys |  |  |  |  |  |  | 1 |  |  |  | 1 | 2 |
| Gln | 2 |  |  |  |  |  | 1 | 1 |  |  | 1 | 5 |
| Ile |  |  |  |  |  |  | 1 | 1 |  |  |  | 2 |
| Gly | 2 |  |  |  |  |  | 2 | 2 |  |  | 2 | 8 |
| Glu | 2 | 1 |  |  |  | 1 | 1 | 1 |  |  | 1 | 7 |
| His |  |  |  |  |  |  | 1 |  |  |  | 1 | 2 |
| Leu | 2 |  |  |  |  |  | 1 | 2 |  |  | 2 | 7 |
| Lys | 2 | 1 |  |  |  |  | 1 | 1 |  |  |  | 5 |
| Met |  |  |  |  |  | 1 | 2 | 2 |  |  |  | 5 |
| Phe |  | 1 |  |  |  |  | 1 | 1 |  |  | 1 | 4 |
| Pro | 1 |  |  |  |  |  | 1 | 1 |  |  |  | 3 |
| SeC |  |  |  |  |  |  |  |  |  |  | 1 | 1 |
| Ser | 1 |  |  |  | 1 |  | 1 | 2 |  |  | 1 | 6 |
| Thr | 2 |  |  |  |  |  |  |  |  |  | 1 | 3 |
| Trp |  |  |  |  |  |  | 1 |  |  |  | 1 | 2 |
| Tyr | 1 |  |  |  |  |  | 1 |  |  |  | 1 | 3 |
| Val | 1 |  |  |  |  |  | 1 | 1 |  |  | 2 | 5 |
| fMet |  |  | 1 |  |  |  |  |  |  |  | 1 | 2 |
| Total | 21 | 6 | 2 | 0 | 1 | 2 | 20 | 22 | 0 | 0 | 20 | 94 |

**Table S4** Numbers of 16S-rRNAs in HI03-3b genome sequence based on KofamKOALA (Aramaki et al., 2020)

| Top Hit | Accession | Contig | | | | | | | | | | | Total |
| --- | --- | --- | --- | --- | --- | --- | --- | --- | --- | --- | --- | --- | --- |
| (BlastN) | number | 1 | 6 | 5 | 4 | 3 | 2 | 14 | 13 | 7 | 8 | 12 |  |
| *C. firmus* NBRC 15306 | NR_112635.1 | 1 | 2 |  | 1 | 1 | 1 | 1 | 1 |  | 1 | 0 | 9 |
| *C. firmus* IAM 12464 | NR_025842.1 |  |  | 2 |  |  |  |  |  |  |  |  | 2 |
| *B. sanguinis* BML-BC004 | NR_175555.1 |  |  |  |  |  |  |  |  | 1 |  |  | 1 |
|  |  |  |  |  |  |  |  |  |  |  |  |  | 12 |

**Table S5** Predicted carbohydrate metabolisms in HI03-3b based on KofamKOALA (Aramaki et al., 2020)

- 1. Central carbohydrate metabolism
     - [M00001](https://www.genome.jp/kegg-bin/show_module?164338224690713/M00001.args.multi) Glycolysis (Embden-Meyerhof pathway), glucose => pyruvate ([10](javascript:display('M00001')))  (complete 9/9)
     - [M00002](https://www.genome.jp/kegg-bin/show_module?164338224690713/M00002.args.multi) Glycolysis, core module involving three-carbon compounds ([6](javascript:display('M00002')))  (complete 5/5)
     - [M00003](https://www.genome.jp/kegg-bin/show_module?164338224690713/M00003.args.multi) Gluconeogenesis, oxaloacetate => fructose-6P ([8](javascript:display('M00003')))  (complete 7/7)
     - [M00009](https://www.genome.jp/kegg-bin/show_module?164338224690713/M00009.args.multi) Citrate cycle (TCA cycle, Krebs cycle) ([17](javascript:display('M00009')))  (complete 8/8)
     - [M00010](https://www.genome.jp/kegg-bin/show_module?164338224690713/M00010.args.multi) Citrate cycle, first carbon oxidation, oxaloacetate => 2-oxoglutarate ([3](javascript:display('M00010')))  (complete 3/3)
     - [M00011](https://www.genome.jp/kegg-bin/show_module?164338224690713/M00011.args.multi) Citrate cycle, second carbon oxidation, 2-oxoglutarate => oxaloacetate ([14](javascript:display('M00011')))  (complete 5/5)
     - [M00004](https://www.genome.jp/kegg-bin/show_module?164338224690713/M00004.args.multi) Pentose phosphate pathway (Pentose phosphate cycle) ([9](javascript:display('M00004')))  (complete 7/7)
     - [M00006](https://www.genome.jp/kegg-bin/show_module?164338224690713/M00006.args.multi) Pentose phosphate pathway, oxidative phase, glucose 6P => ribulose 5P ([3](javascript:display('M00006')))  (complete 2/2)
     - [M00007](https://www.genome.jp/kegg-bin/show_module?164338224690713/M00007.args.multi) Pentose phosphate pathway, non-oxidative phase, fructose 6P => ribose 5P ([5](javascript:display('M00007')))  (complete 4/4)
     - [M00580](https://www.genome.jp/kegg-bin/show_module?164338224690713/M00580.args.multi) Pentose phosphate pathway, archaea, fructose 6P => ribose 5P ([3](javascript:display('M00580')))  (complete 2/2)
     - [M00005](https://www.genome.jp/kegg-bin/show_module?164338224690713/M00005.args.multi) PRPP biosynthesis, ribose 5P => PRPP ([1](javascript:display('M00005')))  (complete 1/1)
     - [M00008](https://www.genome.jp/kegg-bin/show_module?164338224690713/M00008.args.multi) Entner-Doudoroff pathway, glucose-6P => glyceraldehyde-3P + pyruvate ([3](javascript:display('M00008')))  (1 block missing 3/4)
     - [M00308](https://www.genome.jp/kegg-bin/show_module?164338224690713/M00308.args.multi) Semi-phosphorylative Entner-Doudoroff pathway, gluconate => glycerate-3P ([4](javascript:display('M00308')))  (1 block missing 3/4)
  2. Other carbohydrate metabolism
     - [M00854](https://www.genome.jp/kegg-bin/show_module?164338224690713/M00854.args.multi) Glycogen biosynthesis, glucose-1P => glycogen/starch ([5](javascript:display('M00854')))  (complete 2/2)
     - [M00855](https://www.genome.jp/kegg-bin/show_module?164338224690713/M00855.args.multi) Glycogen degradation, glycogen => glucose-6P ([3](javascript:display('M00855')))  (1 block missing 2/3)
     - [M00549](https://www.genome.jp/kegg-bin/show_module?164338224690713/M00549.args.multi) Nucleotide sugar biosynthesis, glucose => UDP-glucose ([3](javascript:display('M00549')))  (complete 3/3)
     - [M00909](https://www.genome.jp/kegg-bin/show_module?164338224690713/M00909.args.multi) UDP-N-acetyl-D-glucosamine biosynthesis, prokaryotes, glucose => UDP-GlcNAc ([5](javascript:display('M00909')))  (complete 5/5)
     - [M00012](https://www.genome.jp/kegg-bin/show_module?164338224690713/M00012.args.multi) Glyoxylate cycle ([5](javascript:display('M00012')))  (complete 5/5)
     - [M00741](https://www.genome.jp/kegg-bin/show_module?164338224690713/M00741.args.multi) Propanoyl-CoA metabolism, propanoyl-CoA => succinyl-CoA ([3](javascript:display('M00741')))  (1 block missing 2/3)

**Table S6** Energy metabolisms in HI03-3b based on KofamKOALA (Aramaki et al., 2020) analysis

2.1. Carbon fixation

- - - [M00167](https://www.genome.jp/kegg-bin/show_module?164338224690713/M00167.args.multi) Reductive pentose phosphate cycle, glyceraldehyde-3P => ribulose-5P ([5](javascript:display('M00167')))  (1 block missing 6/7)
    - [M00168](https://www.genome.jp/kegg-bin/show_module?164338224690713/M00168.args.multi) CAM (Crassulacean acid metabolism), dark ([1](javascript:display('M00168')))  (1 block missing 1/2)
    - [M00169](https://www.genome.jp/kegg-bin/show_module?164338224690713/M00169.args.multi) CAM (Crassulacean acid metabolism), light ([1](javascript:display('M00169')))  (1 block missing 1/2)
    - [M00579](https://www.genome.jp/kegg-bin/show_module?164338224690713/M00579.args.multi) Phosphate acetyltransferase-acetate kinase pathway, acetyl-CoA => acetate ([2](javascript:display('M00579')))  (complete 2/2)

2.2. Methane metabolism

- - - [M00345](https://www.genome.jp/kegg-bin/show_module?164338224690713/M00345.args.multi) Formaldehyde assimilation, ribulose monophosphate pathway ([4](javascript:display('M00345')))  (complete 3/3)

2.3. Sulfur metabolism

- - - [M00176](https://www.genome.jp/kegg-bin/show_module?164338224690713/M00176.args.multi) Assimilatory sulfate reduction, sulfate => H2S ([5](javascript:display('M00176')))  (complete 3/3) ATP synthesis
    - [M00151](https://www.genome.jp/kegg-bin/show_module?164338224690713/M00151.args.multi) Cytochrome bc1 complex respiratory unit ([3](javascript:display('M00151')))  (complete 1/1)
    - [M00416](https://www.genome.jp/kegg-bin/show_module?164338224690713/M00416.args.multi) Cytochrome aa3-600 menaquinol oxidase ([4](javascript:display('M00416')))  (complete 1/1)
    - [M00157](https://www.genome.jp/kegg-bin/show_module?164338224690713/M00157.args.multi) F-type ATPase, prokaryotes and chloroplasts ([8](javascript:display('M00157')))  (complete 1/1)

**Table S7** Lipid metabolisms in HI03-3b based on KofamKOALA (Aramaki et al., 2020) analysis

3.1. Fatty acid metabolism

- - - [M00082](https://www.genome.jp/kegg-bin/show_module?164338224690713/M00082.args.multi) Fatty acid biosynthesis, initiation ([6](javascript:display('M00082')))  (complete 2/2)
    - [M00083](https://www.genome.jp/kegg-bin/show_module?164338224690713/M00083.args.multi) Fatty acid biosynthesis, elongation ([6](javascript:display('M00083')))  (complete 1/1)
    - [M00086](https://www.genome.jp/kegg-bin/show_module?164338224690713/M00086.args.multi) beta-Oxidation, acyl-CoA synthesis ([1](javascript:display('M00086')))  (complete 1/1)
    - [M00087](https://www.genome.jp/kegg-bin/show_module?164338224690713/M00087.args.multi) beta-Oxidation ([5](javascript:display('M00087')))  (complete 3/3) Lipid metabolism
    - [M00098](https://www.genome.jp/kegg-bin/show_module?164338224690713/M00098.args.multi) Acylglycerol degradation ([2](javascript:display('M00098')))  (complete 2/2)
    - [M00093](https://www.genome.jp/kegg-bin/show_module?164338224690713/M00093.args.multi) Phosphatidylethanolamine (PE) biosynthesis, PA => PS => PE ([3](javascript:display('M00093')))  (complete 3/3)

**Table S8** Nucleotide metabolisms in HI03-3b based on KofamKOALA (Aramaki et al., 2020) analysis

4.1. Purine metabolism

- - - [M00049](https://www.genome.jp/kegg-bin/show_module?164338224690713/M00049.args.multi) Adenine ribonucleotide biosynthesis, IMP => ADP, ATP ([4](javascript:display('M00049')))  (complete 4/4)
    - [M00050](https://www.genome.jp/kegg-bin/show_module?164338224690713/M00050.args.multi) Guanine ribonucleotide biosynthesis, IMP => GDP, GTP ([4](javascript:display('M00050')))  (complete 4/4) Pyrimidine metabolism
    - [M00051](https://www.genome.jp/kegg-bin/show_module?164338224690713/M00051.args.multi) Uridine monophosphate biosynthesis, glutamine (+ PRPP) => UMP ([7](javascript:display('M00051')))  (1 block missing 2/3)
    - [M00052](https://www.genome.jp/kegg-bin/show_module?164338224690713/M00052.args.multi) Pyrimidine ribonucleotide biosynthesis, UMP => UDP/UTP, CDP/CTP ([3](javascript:display('M00052')))  (complete 3/3)
    - [M00053](https://www.genome.jp/kegg-bin/show_module?164338224690713/M00053.args.multi) Pyrimidine deoxyribonuleotide biosynthesis, CDP => dCTP ([3](javascript:display('M00053')))  (complete 2/2)
    - [M00938](https://www.genome.jp/kegg-bin/show_module?164338224690713/M00938.args.multi) Pyrimidine deoxyribonuleotide biosynthesis, UDP => dTTP ([5](javascript:display('M00938')))  (complete 5/5)

**Table S9** Predicted amino acid metabolisms in HI03-3b based on KofamKOALA (Aramaki et al., 2020)

- 1. Serine and threonine metabolisms
     - [M00020](https://www.genome.jp/kegg-bin/show_module?164338224690713/M00020.args.multi) Serine biosynthesis, glycerate-3P => serine ([2](javascript:display('M00020')))  (1 block missing 2/3)
     - [M00018](https://www.genome.jp/kegg-bin/show_module?164338224690713/M00018.args.multi) Threonine biosynthesis, aspartate => homoserine => threonine ([5](javascript:display('M00018')))  (complete 5/5)
  2. Cysteine and methionine metabolism
     - [M00021](https://www.genome.jp/kegg-bin/show_module?164338224690713/M00021.args.multi) Cysteine biosynthesis, serine => cysteine ([2](javascript:display('M00021')))  (complete 2/2)
     - [M00609](https://www.genome.jp/kegg-bin/show_module?164338224690713/M00609.args.multi) Cysteine biosynthesis, methionine => cysteine ([6](javascript:display('M00609')))  (complete 6/6)
     - [M00017](https://www.genome.jp/kegg-bin/show_module?164338224690713/M00017.args.multi) Methionine biosynthesis, aspartate => homoserine => methionine ([9](javascript:display('M00017')))  (complete 7/7)
  3. Branched-chain amino acid metabolism
     - [M00019](https://www.genome.jp/kegg-bin/show_module?164338224690713/M00019.args.multi) Valine/isoleucine biosynthesis, pyruvate => valine / 2-oxobutanoate => isoleucine ([5](javascript:display('M00019')))  (complete 4/4)
     - [M00535](https://www.genome.jp/kegg-bin/show_module?164338224690713/M00535.args.multi) Isoleucine biosynthesis, pyruvate => 2-oxobutanoate ([3](javascript:display('M00535')))  (1 block missing 2/3)
     - [M00570](https://www.genome.jp/kegg-bin/show_module?164338224690713/M00570.args.multi) Isoleucine biosynthesis, threonine => 2-oxobutanoate => isoleucine ([6](javascript:display('M00570')))  (complete 5/5)
     - [M00432](https://www.genome.jp/kegg-bin/show_module?164338224690713/M00432.args.multi) Leucine biosynthesis, 2-oxoisovalerate => 2-oxoisocaproate ([4](javascript:display('M00432')))  (complete 3/3)
  4. Lysine metabolism
     - [M00525](https://www.genome.jp/kegg-bin/show_module?164338224690713/M00525.args.multi) Lysine biosynthesis, acetyl-DAP pathway, aspartate => lysine ([9](javascript:display('M00525')))  (complete 9/9)
     - [M00526](https://www.genome.jp/kegg-bin/show_module?164338224690713/M00526.args.multi) Lysine biosynthesis, DAP dehydrogenase pathway, aspartate => lysine ([5](javascript:display('M00526')))  (1 block missing 5/6)
     - [M00527](https://www.genome.jp/kegg-bin/show_module?164338224690713/M00527.args.multi) Lysine biosynthesis, DAP aminotransferase pathway, aspartate => lysine ([6](javascript:display('M00527')))  (1 block missing 6/7)
  5. Arginine and proline metabolism
     - [M00028](https://www.genome.jp/kegg-bin/show_module?164338224690713/M00028.args.multi) Ornithine biosynthesis, glutamate => ornithine ([4](javascript:display('M00028')))  (1 block missing 3/4)
     - [M00844](https://www.genome.jp/kegg-bin/show_module?164338224690713/M00844.args.multi) Arginine biosynthesis, ornithine => arginine ([3](javascript:display('M00844')))  (complete 3/3)
     - [M00029](https://www.genome.jp/kegg-bin/show_module?164338224690713/M00029.args.multi) Urea cycle ([4](javascript:display('M00029')))  (1 block missing 4/5)
     - [M00015](https://www.genome.jp/kegg-bin/show_module?164338224690713/M00015.args.multi) Proline biosynthesis, glutamate => proline ([3](javascript:display('M00015')))  (complete 2/2)
  6. Polyamine biosynthesis

- - - [M00133](https://www.genome.jp/kegg-bin/show_module?164338224690713/M00133.args.multi) Polyamine biosynthesis, arginine => agmatine => putrescine => spermidine ([4](javascript:display('M00133')))  (complete 4/4)
    - [M00134](https://www.genome.jp/kegg-bin/show_module?164338224690713/M00134.args.multi) Polyamine biosynthesis, arginine => ornithine => putrescine ([1](javascript:display('M00134')))  (1 block missing 1/2)
  1. Histidine metabolism
     - [M00026](https://www.genome.jp/kegg-bin/show_module?164338224690713/M00026.args.multi) Histidine biosynthesis, PRPP => histidine ([10](javascript:display('M00026')))  (complete 6/6)
     - [M00045](https://www.genome.jp/kegg-bin/show_module?164338224690713/M00045.args.multi) Histidine degradation, histidine => *N*-formiminoglutamate => glutamate ([4](javascript:display('M00045')))  (complete 4/4)
  2. Aromatic amino acid metabolism
     - [M00022](https://www.genome.jp/kegg-bin/show_module?164338224690713/M00022.args.multi) Shikimate pathway, phosphoenolpyruvate + erythrose-4P => chorismate ([7](javascript:display('M00022')))  (complete 4/4)
     - [M00023](https://www.genome.jp/kegg-bin/show_module?164338224690713/M00023.args.multi) Tryptophan biosynthesis, chorismate => tryptophan ([7](javascript:display('M00023')))  (complete 3/3)
     - [M00024](https://www.genome.jp/kegg-bin/show_module?164338224690713/M00024.args.multi) Phenylalanine biosynthesis, chorismate => phenylpyruvate => phenylalanine ([3](javascript:display('M00024')))  (1 block missing 1/2)
     - [M00025](https://www.genome.jp/kegg-bin/show_module?164338224690713/M00025.args.multi) Tyrosine biosynthesis, chorismate => HPP => tyrosine ([3](javascript:display('M00025')))  (1 block missing 1/2)
  3. Other amino acid metabolism
     - [M00027](https://www.genome.jp/kegg-bin/show_module?164338224690713/M00027.args.multi) GABA (gamma-Aminobutyrate) shunt ([2](javascript:display('M00027')))  (1 block missing 2/3)

**Table S10** Predicted glycan metabolism in HI03-3b based on KofamKOALA (Aramaki et al., 2020)

6.1. Lipopolysaccharide metabolism

- - - [M00923](https://www.genome.jp/kegg-bin/show_module?164338224690713/M00923.args.multi) UDP-L-FucNAm biosynthesis ([3](javascript:display('M00923')))  (1 block missing 3/4)

**Table S11** Carbon and vitamins metabolisms in HI03-3b based on KofamKOALA (Aramaki et al., 2020)

7.1. Cofactor and vitamin metabolism

- - - [M00899](https://www.genome.jp/kegg-bin/show_module?164338224690713/M00899.args.multi) Thiamine salvage pathway, HMP/HET => TMP ([3](javascript:display('M00899')))  (complete 2/2)
    - [M00125](https://www.genome.jp/kegg-bin/show_module?164338224690713/M00125.args.multi) Riboflavin biosynthesis, plants and bacteria, GTP => riboflavin/FMN/FAD ([7](javascript:display('M00125')))  (complete 7/7)
    - [M00916](https://www.genome.jp/kegg-bin/show_module?164338224690713/M00916.args.multi) Pyridoxal-P biosynthesis, R5P + glyceraldehyde-3P + glutamine => pyridoxal-P ([2](javascript:display('M00916')))  (complete 1/1)
    - [M00115](https://www.genome.jp/kegg-bin/show_module?164338224690713/M00115.args.multi) NAD biosynthesis, aspartate => quinolinate => NAD ([6](javascript:display('M00115')))  (complete 5/5)
    - [M00119](https://www.genome.jp/kegg-bin/show_module?164338224690713/M00119.args.multi) Pantothenate biosynthesis, valine/L-aspartate => pantothenate ([5](javascript:display('M00119')))  (complete 5/5)
    - [M00913](https://www.genome.jp/kegg-bin/show_module?164338224690713/M00913.args.multi) Pantothenate biosynthesis, 2-oxoisovalerate/spermine => pantothenate ([4](javascript:display('M00913')))  (1 block missing 4/5)
    - [M00120](https://www.genome.jp/kegg-bin/show_module?164338224690713/M00120.args.multi) Coenzyme A biosynthesis, pantothenate => CoA ([4](javascript:display('M00120')))  (1 block missing 2/3)
    - [M00123](https://www.genome.jp/kegg-bin/show_module?164338224690713/M00123.args.multi) Biotin biosynthesis, pimeloyl-ACP/CoA => biotin ([5](javascript:display('M00123')))  (complete 3/3)
    - [M00573](https://www.genome.jp/kegg-bin/show_module?164338224690713/M00573.args.multi) Biotin biosynthesis, BioI pathway, long-chain-acyl-ACP => pimeloyl-ACP => biotin ([4](javascript:display('M00573')))  (1 block missing 4/5)
    - [M00577](https://www.genome.jp/kegg-bin/show_module?164338224690713/M00577.args.multi) Biotin biosynthesis, BioW pathway, pimelate => pimeloyl-CoA => biotin ([5](javascript:display('M00577')))  (1 block missing 4/5)
    - [M00881](https://www.genome.jp/kegg-bin/show_module?164338224690713/M00881.args.multi) Lipoic acid biosynthesis, plants and bacteria, octanoyl-ACP => dihydrolipoyl-E2/H ([1](javascript:display('M00881')))  (1 block missing 1/2)
    - [M00882](https://www.genome.jp/kegg-bin/show_module?164338224690713/M00882.args.multi) Lipoic acid biosynthesis, eukaryotes, octanoyl-ACP => dihydrolipoyl-H ([1](javascript:display('M00882')))  (1 block missing 1/2)
    - [M00883](https://www.genome.jp/kegg-bin/show_module?164338224690713/M00883.args.multi) Lipoic acid biosynthesis, animals and bacteria, octanoyl-ACP => dihydrolipoyl-H => dihydrolipoyl-E2 ([3](javascript:display('M00883')))  (complete 3/3)
    - [M00884](https://www.genome.jp/kegg-bin/show_module?164338224690713/M00884.args.multi) Lipoic acid biosynthesis, octanoyl-CoA => dihydrolipoyl-E2 ([1](javascript:display('M00884')))  (1 block missing 1/2)
    - [M00126](https://www.genome.jp/kegg-bin/show_module?164338224690713/M00126.args.multi) Tetrahydrofolate biosynthesis, GTP => THF ([7](javascript:display('M00126')))  (1 block missing 4/5)
    - [M00842](https://www.genome.jp/kegg-bin/show_module?164338224690713/M00842.args.multi) Tetrahydrobiopterin biosynthesis, GTP => BH4 ([2](javascript:display('M00842')))  (1 block missing 2/3)
    - [M00843](https://www.genome.jp/kegg-bin/show_module?164338224690713/M00843.args.multi) L-threo-Tetrahydrobiopterin biosynthesis, GTP => L-threo-BH4 ([2](javascript:display('M00843')))  (1 block missing 2/3)
    - [M00880](https://www.genome.jp/kegg-bin/show_module?164338224690713/M00880.args.multi) Molybdenum cofactor biosynthesis, GTP => molybdenum cofactor ([5](javascript:display('M00880')))  (complete 3/3)
    - [M00140](https://www.genome.jp/kegg-bin/show_module?164338224690713/M00140.args.multi) C1-unit interconversion, prokaryotes ([3](javascript:display('M00140')))  (complete 3/3)
    - [M00141](https://www.genome.jp/kegg-bin/show_module?164338224690713/M00141.args.multi) C1-unit interconversion, eukaryotes ([1](javascript:display('M00141')))  (1 block missing 1/2)
    - [M00846](https://www.genome.jp/kegg-bin/show_module?164338224690713/M00846.args.multi) Siroheme biosynthesis, glutamyl-tRNA => siroheme ([7](javascript:display('M00846')))  (1 block missing 5/6)
    - [M00121](https://www.genome.jp/kegg-bin/show_module?164338224690713/M00121.args.multi) Heme biosynthesis, plants and bacteria, glutamate => heme ([10](javascript:display('M00121')))  (complete 10/10)
    - [M00926](https://www.genome.jp/kegg-bin/show_module?164338224690713/M00926.args.multi) Heme biosynthesis, bacteria, glutamyl-tRNA => coproporphyrin III => heme ([9](javascript:display('M00926')))  (complete 9/9)
    - [M00924](https://www.genome.jp/kegg-bin/show_module?164338224690713/M00924.args.multi) Cobalamin biosynthesis, anaerobic, uroporphyrinogen III => sirohydrochlorin => cobyrinate a, c-diamide ([13](javascript:display('M00924')))  (complete 11/11)
    - [M00122](https://www.genome.jp/kegg-bin/show_module?164338224690713/M00122.args.multi) Cobalamin biosynthesis, cobyrinate a, c-diamide => cobalamin ([7](javascript:display('M00122')))  (1 block missing 6/7)
    - [M00116](https://www.genome.jp/kegg-bin/show_module?164338224690713/M00116.args.multi) Menaquinone biosynthesis, chorismate (+ polyprenyl-PP) => menaquinol ([8](javascript:display('M00116')))  (1 block missing 8/9)

**Table S12** Predicted terpenoid biosynthesis in HI03-3b based on KofamKOALA (Aramaki et al., 2020)

8.1. Terpenoid backbone biosynthesis

- - - [M00096](https://www.genome.jp/kegg-bin/show_module?164338224690713/M00096.args.multi) C5 isoprenoid biosynthesis, non-mevalonate pathway ([8](javascript:display('M00096')))  (complete 8/8)
    - [M00364](https://www.genome.jp/kegg-bin/show_module?164338224690713/M00364.args.multi) C10-C20 isoprenoid biosynthesis, bacteria ([2](javascript:display('M00364')))  (complete 2/2)
    - [M00365](https://www.genome.jp/kegg-bin/show_module?164338224690713/M00365.args.multi) C10-C20 isoprenoid biosynthesis, archaea ([1](javascript:display('M00365')))  (1 block missing 1/2)

**Table S13** some genes encoding carbohydrate active enzymes

| **Gene ID** | **AA** | **KEGG Orthology search** | **BLASTx search** | | **Proposed function** |
| --- | --- | --- | --- | --- | --- |
|  |  |  | **Protein** | **I/S (%)** |  |
| HI03-512 | 558 | oligo-1,6-glucosidase/ α-glucosidase [EC:3.2.1.10] | α-glucosidase [WP_222497254.1] | 99/99 | α-glucosidase |
| HI03-513 | 561 | trehalose-6-phosphate hydrolase [EC:3.2.1.93] | α-phosphotrehalase [WP_222497253.1] | 99/99 | α-phosphotrehalase |
| HI03-1147 | 561 | oligo-1,6-glucosidase [EC:3.2.1.10] | alpha-glucosidase [WP_197246158.1] | 99/99 | α-glucosidase |
| HI03-1148 | 539 | oligo-1,6-glucosidase [EC:3.2.1.10] | alpha-glucosidase [WP_262172852.1] | 99/100 | α-glucosidase |
| HI03-2057 | 496 | β-fructofuranosidase [EC:3.2.1.26] | sucrose-6-phosphate hydrolase [WP_252218572.1] | 97/98 | sucrose-6-phosphate hydrolase |
| HI03-5125 | 1120 | mannosyl-glycoprotein endo-β-*N*-acetylglucosaminidase [EC:3.2.1.96] | *N*-acetylglucosaminidase [WP_139344984.1] | 41/57 | *N*-acetylglucosaminidase |
| HI03-2145 | 222 | β-glucosylceramidase [EC:3.2.1.45] | glycoside hydrolase family 30 protein [MBN8203477.1] | 94/95 | glucosylceramidase |
| HI03-1798 | 443 | xylose isomerase [EC:5.3.1.5] | xylose isomerase [WP_248347596.1] | 100/100 | xylose isomerase |
| HI03-5147 | 417 | L-rhamnose isomerase [EC:5.3.1.14] | L-rhamnose isomerase [WP_258751270.1] | 98/100 | L-rhamnose isomerase |
| HI03-4199 | 308 | purine nucleosidase [EC:3.2.2.1] | nucleoside hydrolase [WP_197214150.1] | 100/100 | nucleoside hydrolase |
| HI03-424 | 213 | soluble lytic murein transglycosylase [EC:4.2.2.-] | lytic transglycosylase domain-containing protein [WP_197214652.1] | 100/100 | lytic transglycosylase |
| HI03-1355 | 387 | chitinase [EC:3.2.1.14] | glycosyl hydrolase family 18 [WP_227887042.1] | 98/99 | chitinase |
| HI03-1798 | 443 | xylose isomerase [EC:5.3.1.5] | xylose isomerase [WP_227887452.1] | 99/99 | xylose isomerase |
| HI03-2670 | 521 | pullulanase [EC:3.2.1.41] | type I pullulanase [WP_222500182.1] | 99/  99 | pullulanase |

**Table S14** Genes on HI03-3b genome encoding biotechnologically potential enzymes

| **Gene ID** | **AA** | **KEGG Orthology search**  (Aramaki et al., 2020) | | **BLASTx search** (Altschul et al., 1990) | | | **Proposed function** |
| --- | --- | --- | --- | --- | --- | --- | --- |
|  |  | KO/  HMM | Protein | Protein | Organism | I/S (%) |  |
| HI03-104 | 301 | K01476  369.3 | arginase [EC:3.5.3.1] | arginase  [WP_197201066.1] | *Cytobacillus firmus* | 99/  100 | arginase |
| HI03-309 | 214 | K13282  116.9 | cyanophycinase [EC:3.4.15.6] | cyanophycinase  [SUV02533.1] | *C. firmus* | 89/  95 | cyanophycinase |
| HI03-512 | 558 | K01187 277.10 | alpha-glucosidase [EC:3.2.1.20] | alpha-glucosidase WP_222497254.1 | *C. firmus* | 99/  99 | α-glucosidase |
| HI03-932 | 411 | K07263  287.1 | zinc protease [EC:3.4.24.-] | insulinase family [WP_227887042.1] | *C. firmus* | 99/  100 | zinc-dependent metallo-proteinase |
| HI03-1355 | 387 | K01183  132.3 | chitinase [EC:3.2.1.14] | glycosyl hydrolase family 18 [WP_227887042.1] | *Cytobacillus* sp. | 98/  99 | chitinase |
| HI03-1694 | 593 | K01400  565.2 | bacillolysin [EC:3.4.24.28] | M4 family metallopeptidase  [WP_222500569.1] | *C. firmus* | 99/  99 | bacillolysin |
| HI03-1798 | 443 | K01805  691.1 | xylose isomerase [EC:5.3.1.5] | xylose isomerase  [WP_227887452.1] | *C. oceanisediminis* | 99/  99 | xylose isomerase |
| HI03-2166 | 449 | K17734  397.3 | serine protease AprX [EC:3.4.21.-] | S8 family peptidase [WP_226617693.1] | *C. firmus* | 99/  99 | serine protease |
| HI03-2463 | 348 | K13953  507.3 | ADH, propanol-preferring [EC:1.1.1.1] | alcohol dehydrogenase AdhP [WP_227888089.1] | *C. oceanisediminis* | 99/  99 | alcohol dehydrogenase |
| HI03-2670 | 521 | K01200  594.8 | pullulanase [EC:3.2.1.41] | type I pullulanase [WP_222500182.1] | *C. firmus* | 99/  99 | pullulanase |
| HI03-3302 | 306 | K01424  316.1 | L-asparaginase [EC:3.5.1.1] | Asparaginase  [WP_222497870.1] | *C. firmus* | 100/100 | L-asparaginase |
| HI03-3438 | 958 | K01200  984.7 | pullulanase [EC:3.2.1.41] | type I pullulanase [WP_222500182.1] | *C. firmus* | 99/  99 | pullulanase |
| HI03-3561 | 391 | K20486  401.2 | lantibiotic leader peptide-processing serine protease [EC:3.4.21.-] | S8 family serine peptidase [WP_048011423.1] | *C. firmus* | 99/  99 | subtilisin |
| HI03-3984 | 399 | K04771  292.0 | serine protease Do [EC:3.4.21.107] | trypsin-like peptidase domain-containing protein  [WP_222498307.1] | *C. firmus* | 100/  100 | trypsin-like serine peptidase |
| HI03-2429 | 200 | K06999 | carboxylesterase [EC 3.1.1.1] | carboxylesterase  [OMF59661.1] | *Paenibacillus* sp. FSL R5-0490 | 96/  99 | carboxylesterase |
| HI03-2637 | 267 | K01054 | lysophospholipase [EC:3.1.1.5] | phospholipase [OMF54228.1] | *Paenibacillus sp.* FSL R5-0490 | 99/  100 | lysophospholipase |
| HI03-63 | 261 | K17836 | beta-lactamase class A [EC:3.5.2.6] | class A beta-lactamase-related serine hydrolase [WP_226620288.1] | *C. firmus* | 95/  97 | β-lactamase |
| HI03-3504 | 425 | K01438 | acetylornithine deacetylase [EC:3.5.1.16] | acetylornithine deacetylase [OMF56067.1] | *Paenibacillus* sp. FSL R5-0490 | 98/  99 | acetylornithine deacetylase |
| HI03-4153 | 346 | K13979 | alcohol dehydrogenase (NADP+) [EC:1.1.1.2] | NAD(P)-dep. alcohol dehydrogenase [WP_227888799.1] | *C. oceanisediminis* | 99/  99 | NADP-dep. ADH [WP_248349163.1] |
| HI03-4410 | 296 | K01585 | arginine decarboxylase [EC:4.1.1.19] | arginine decarboxylase [PAE22928.1] | *Bacillus* sp. 7894-2 | 94/  96 | SpeA_aminotransferase class I [WP_248349356.1] |
| HI03-5147 | 417 | K01813 | L-rhamnose isomerase [EC:5.3.1.14] | L-rhamnose isomerase [WP_258751270.1] |  | 98/  100 | L-rhamnose isomerase |

Abbreviations: KO, KEGG Ortholog; I, identity; S, similarity

Notes:

- **Arginase** has been used for environmentally friendly preparation of L-ornithine as food supplement and nutrition product (Li et al., 2021).
- **Microbial proteases** have found various applications in the detergent, food, pharmaceutical, leather, and textile industries (Rao et al., 1998).
- **Chitinase** has been applied in food industry, medicine, cosmetics, and crop protection (Poria et al., 2021). GH18 chitinase was particularly subjected to protein engineering for the development of tailor-made chitinases (Poria et al., 2021).
- **Xylose isomerase** has widely been used in the production of high fructose corn syrup (HFCS) and is potentially explored to produce some value-added chemicals in the food, cosmetics, and pharmaceutical industries (Miyamoto et al., 2021).
- **L-asparaginase** has been used as anticancer chemotherapeutic agents for the treatment of leukemia, Hodgkin disease, and sarcoma (Egler et al., 2016; Narta et al., 2007; Verma et al., 2007; Wriston, 1985). It is also used to reduce acrylamide levels in cooked carbohydrate-rich food especially during the heat treatment of starchy foods (Jia et al., 2021).
- **Carboxylesterases (CEs)** have been applied in xenobiotic & endobiotic degradations, biocatalysis, & drug metabolism (Wheelock et al., 2005)
- **Phospholipases** have been used in scientific and medical research, such as inhibitors for generating anti-inflammatory agents and as diagnostic markers for microbial infections (Karabina et al., 2010; Sutto-Ortiz et al., 2017).
- **Arginine decarboxylase** catalyzes conversion of l-arginine into agmatine, a valuable pharmaceutical intermediate with various potential therapeutic functions in neurotransmitter systems, nitric oxide synthesis, and polyamine metabolism (Sun et al., 2017).
- **L-rhamnose isomerase** catalyzes the isomerization between L-rhamnose and L-rhamnulose. It has great potential application in producing a wide variety of rare sugars (Xu et al., 2016)


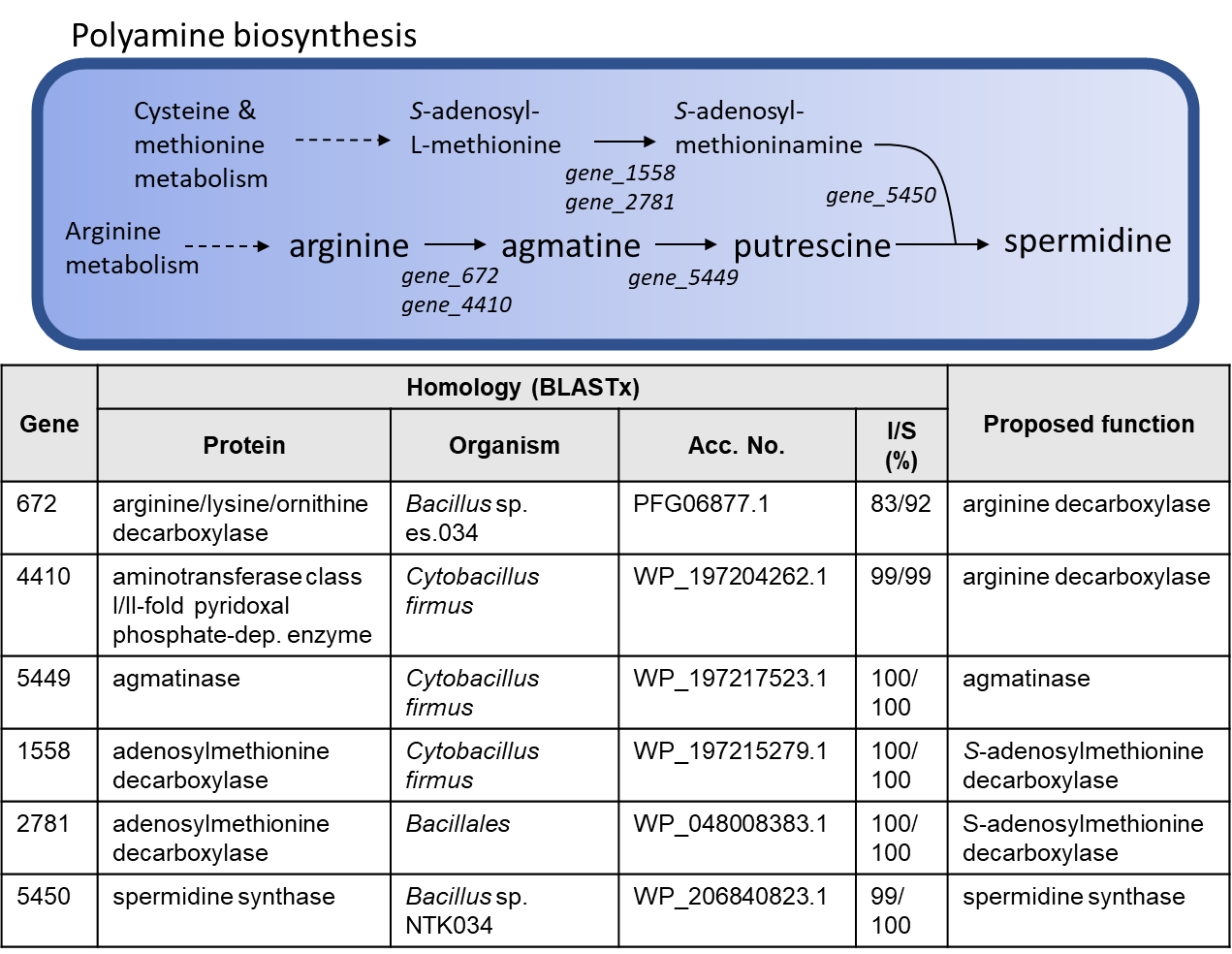


**Figure S4.** Genes involved in spermidine biosynthesis identified in the HI03-3b genome, which were predicted based on KofamKOALA (Aramaki et al., 2020) and BLASTx (Altschul et al., 1990). Notes: spermidine has been shown to increase epithelial renewal and anti-inflammatory macrophage development in the colon, highlighting its importance in the maintenance of intestinal homoeostasis and immunity (Nakamura et al., 2021). From medical perspective, spermidine has been known to extend life span in model organisms, indicating its potential application in delaying aging and promoting longevity in human (Madeo et al., 2018).


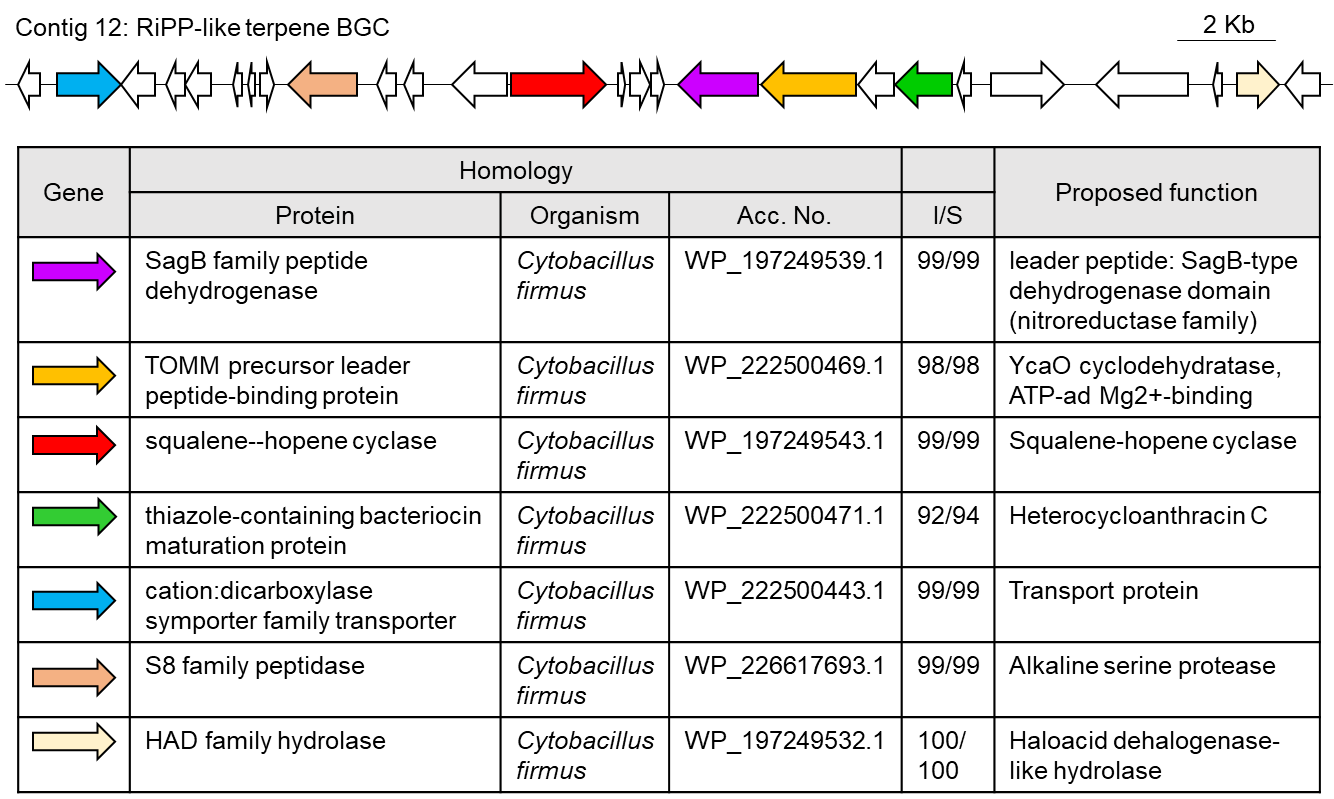


**Figure S5** RiPP-like terpene BGC identified in the HI03-3b genome, which were predicted based on antiSMASH (Blin et al., 2021) and BLASTx (Altschul et al., 1990)


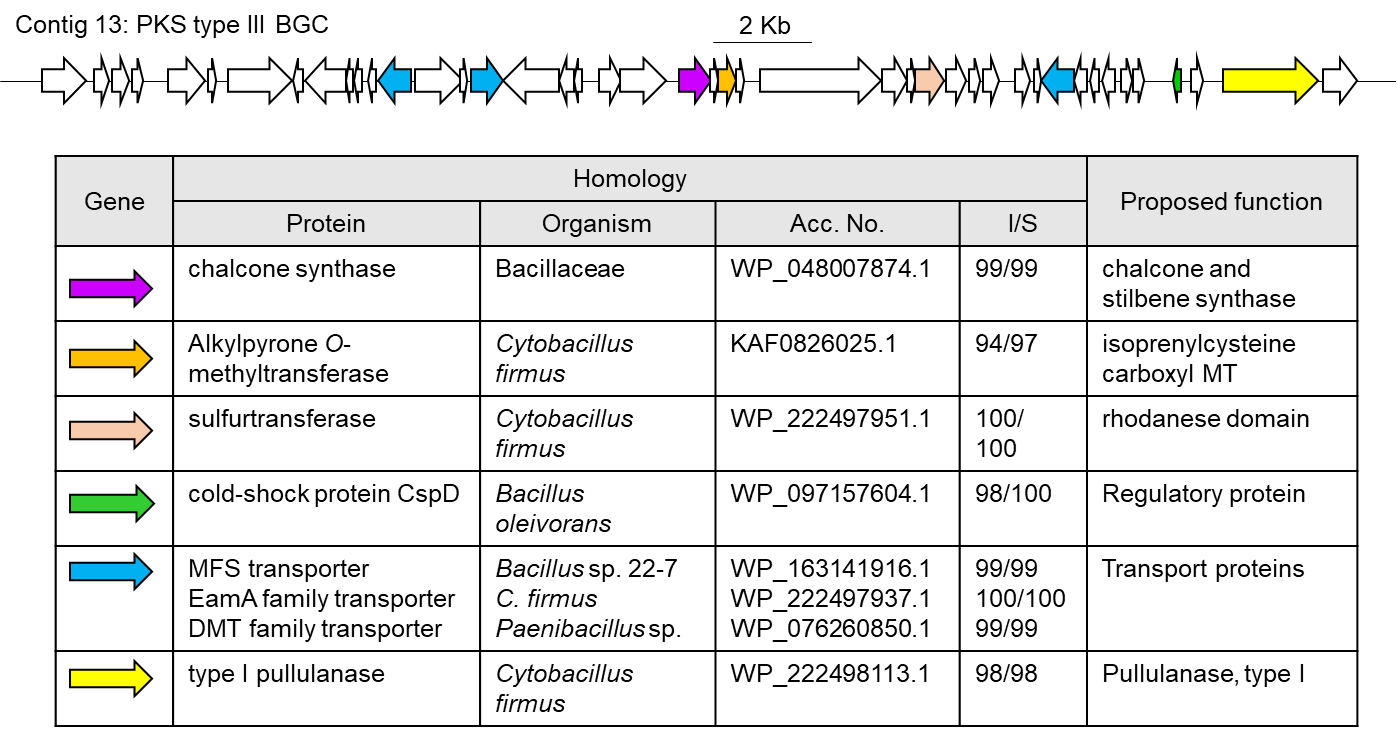


**Figure S6** PKS type III BGC identified in the HI03-3b genome, which were predicted based on antiSMASH (Blin et al., 2021) and BLASTx (Altschul et al., 1990).


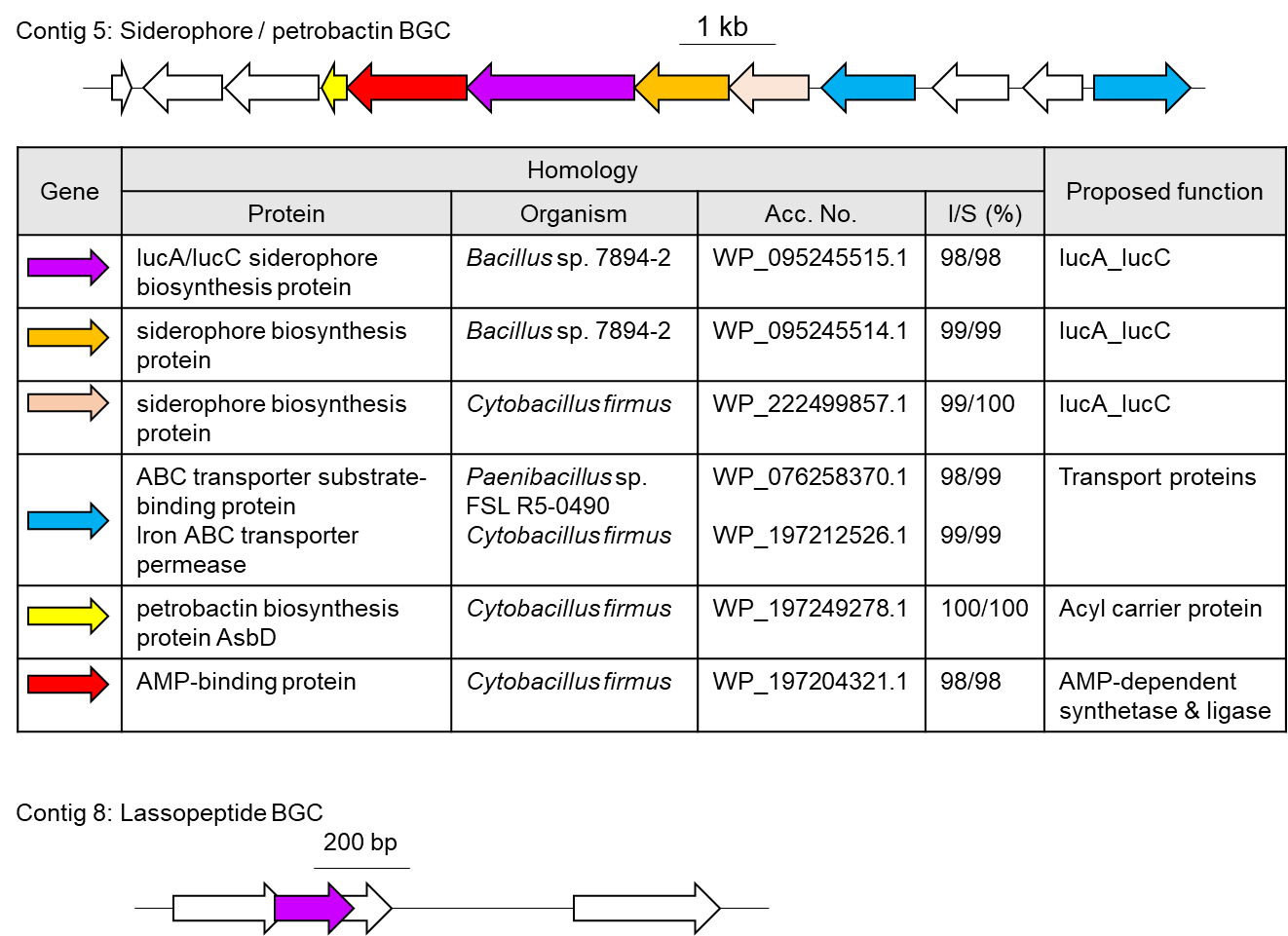


**Figure S7** Siderophore/petrobactin BGC and lassopeptide BGC identified in the HI03-3b genome, which were predicted based on antiSMASH (Blin et al., 2021) and BLASTx (Altschul et al., 1990).

**Table S15** Comparation of biosynthetically potential genes between *Cytobacillus* HI03-3b and *C. firmus* NCTC10335

| Genetically encoded protein or small molecule | Gene presence & copy number | |
| --- | --- | --- |
|  | *Cytobacillus* HI03-3b  [GCA_023197125.1]  (In this work) | *C. firmus* NCTC10335  [GCA_900445365.1] |
| **Carbohydrate active enzymes:** |  |  |
| oligo-1,6-glucosidase [EC:3.2.1.10] | **5** | 4 |
| α-glucosidase [EC:3.2.1.20] | **3** | **1** |
| trehalose-6-phosphate hydrolase [EC:3.2.1.93] | **1** | **1** |
| β-fructofuranosidase [EC:3.2.1.26] | **1** | **1** |
| mannosyl-glycoprotein endo-β-*N*-acetylglucosaminidase [EC:3.2.1.96] | **1** | **-** |
| β-glucosylceramidase [EC:3.2.1.45] | **1** | **-** |
| xylose isomerase [EC:5.3.1.5] | **1** | **-** |
| L-rhamnose isomerase [EC:5.3.1.14] | **1** | **-** |
| purine nucleosidase [EC:3.2.2.1] | **3** | **1** |
| soluble lytic murein transglycosylase [EC:4.2.2.-] | **1** | **1** |
| chitinase [EC:3.2.1.14] | **1** | **-** |
| pullulanase [EC:3.2.1.41] | **2** | **2** |
| neopullulanase [EC:3.2.1.135] | **-** | **2** |
| α-amylase [EC:3.2.1.1] | **-** | **1** |
| **Protein-degrading enzymes:** |  |  |
| zinc protease [EC:3.4.24.-] | **14** | **11** |
| serine protease [EC:3.4.21.-] | **8** | **9** |
| bacillolysin [EC:3.4.24.28] | **2** | **1** |
| **Other enzymes:** |  |  |
| L-asparaginase [EC:3.5.1.1] | **1** | **1** |
| carboxylesterase [EC 3.1.1.1] | **2** | **4** |
| lysophospholipase [EC:3.1.1.5] | **1** | **1** |
| arginase [EC:3.5.3.1] | **1** | **-** |
| dimethylargininase [EC:3.5.3.18] | **1** | **1** |
| arginine decarboxylase [EC:4.1.1.19] | **2** | **2** |
| beta-lactamase class A [EC:3.5.2.6] | **1** | **3** |
| acetylornithine deacetylase [EC:3.5.1.16] | **1** | **2** |
| alcohol dehydrogenase (NADP+) [EC:1.1.1.2] | **1** | **1** |
| **Polyamine biosynthesis:** |  |  |
| arginine => agmatine => putrescine => spermidine | **+** | **+** |
| **Vitamin biosynthesis:** |  |  |
| thiamine (B1) | **+** | **+** |
| riboflavin (B2) | **+** | **+** |
| pantothenate (B5) | **+** | **+** |
| pyridoxine (B6) | **+** | **+** |
| biotin (B7) | **+** | **+** |
| lipoic acid | **+** | **+** |
| cobalamin (B12) | **+** | **+** |
| menaquinone (K2) | **+** | **+** |
| **Secondary metabolite biosynthesis:** |  |  |
| siderophore | **1** | **1** |
| terpene | **1** | **1** |
| type III polyketide synthase (PKS) | **1** | **1** |
| lassopeptide | **1** | **-** |
| lanthipeptide Class II | **-** | **1** |

**Note:** primary metabolisms above predicted based on KofamKOALA (Aramaki et al., 2020), while secondary metabolite biosynthesis based on antiSMASH analysis (Blin et al., 2021).

**References**

Altschul, S.F., Gish, W., Miller, W., Myers, E.W., Lipman, D.J., 1990. Basic local alignment search tool. Journal of Molecular Biology 215, 403–410. https://doi.org/10.1016/S0022-2836(05)80360-2

Aramaki, T., Blanc-Mathieu, R., Endo, H., Ohkubo, K., Kanehisa, M., Goto, S., Ogata, H., 2020. KofamKOALA: KEGG Ortholog assignment based on profile HMM and adaptive score threshold. Bioinformatics 36, 2251–2252. https://doi.org/10.1093/bioinformatics/btz859

Besemer, J., Lomsadze, A., Borodovsky, M., 2001. GeneMarkS: a self-training method for prediction of gene starts in microbial genomes. Implications for finding sequence motifs in regulatory regions. Nucleic Acids Research 29, 2607–2618. https://doi.org/10.1093/nar/29.12.2607

Blin, K., Shaw, S., Kloosterman, A.M., Charlop-Powers, Z., van Wezel, G.P., Medema, M.H., Weber, T., 2021. antiSMASH 6.0: improving cluster detection and comparison capabilities. Nucleic Acids Res 49, W29–W35. https://doi.org/10.1093/nar/gkab335

Chan, P.P., Lin, B.Y., Mak, A.J., Lowe, T.M., 2021. tRNAscan-SE 2.0: improved detection and functional classification of transfer RNA genes. Nucleic Acids Research 49, 9077–9096. https://doi.org/10.1093/nar/gkab688

Darling, A.C.E., Mau, B., Blattner, F.R., Perna, N.T., 2004. Mauve: Multiple Alignment of Conserved Genomic Sequence With Rearrangements. Genome Res 14, 1394–1403. https://doi.org/10.1101/gr.2289704

Egler, R.A., Ahuja, S.P., Matloub, Y., 2016. L-asparaginase in the treatment of patients with acute lymphoblastic leukemia. J Pharmacol Pharmacother 7, 62–71. https://doi.org/10.4103/0976-500X.184769

Gurevich, A., Saveliev, V., Vyahhi, N., Tesler, G., 2013. QUAST: quality assessment tool for genome assemblies. Bioinformatics 29, 1072–1075. https://doi.org/10.1093/bioinformatics/btt086

Jia, R., Wan, X., Geng, X., Xue, D., Xie, Z., Chen, C., 2021. Microbial L-asparaginase for Application in Acrylamide Mitigation from Food: Current Research Status and Future Perspectives. Microorganisms 9, 1659. https://doi.org/10.3390/microorganisms9081659

Karabina, S.-A., Gora, S., Atout, R., Ninio, E., 2010. Extracellular phospholipases in atherosclerosis. Biochimie, Phospholipases A2 and lipid mediators 92, 594–600. https://doi.org/10.1016/j.biochi.2010.02.002

Li, M., Qin, J., Xiong, K., Jiang, B., Zhang, T., 2021. Review of arginase as a promising biocatalyst: characteristics, preparation, applications and future challenges. Critical Reviews in Biotechnology 0, 1–17. https://doi.org/10.1080/07388551.2021.1947962

Lowe, T.M., Chan, P.P., 2016. tRNAscan-SE On-line: integrating search and context for analysis of transfer RNA genes. Nucleic Acids Res 44, W54–W57. https://doi.org/10.1093/nar/gkw413

Luo, C., Rodriguez-R, L.M., Konstantinidis, K.T., 2014. MyTaxa: an advanced taxonomic classifier for genomic and metagenomic sequences. Nucleic Acids Res 42, e73. https://doi.org/10.1093/nar/gku169

Madeo, F., Carmona-Gutierrez, D., Kepp, O., Kroemer, G., 2018. Spermidine delays aging in humans. Aging (Albany NY) 10, 2209–2211. https://doi.org/10.18632/aging.101517

Miyamoto, R.Y., de Melo, R.R., de Mesquita Sampaio, I.L., de Sousa, A.S., Morais, E.R., Sargo, C.R., Zanphorlin, L.M., 2021. Paradigm shift in xylose isomerase usage: a novel scenario with distinct applications. Crit Rev Biotechnol 1–20. https://doi.org/10.1080/07388551.2021.1962241

Nakamura, A., Kurihara, S., Takahashi, D., Ohashi, W., Nakamura, Y., Kimura, S., Onuki, M., Kume, A., Sasazawa, Y., Furusawa, Y., Obata, Y., Fukuda, S., Saiki, S., Matsumoto, M., Hase, K., 2021. Symbiotic polyamine metabolism regulates epithelial proliferation and macrophage differentiation in the colon. Nat Commun 12, 2105. https://doi.org/10.1038/s41467-021-22212-1

Narta, U.K., Kanwar, S.S., Azmi, W., 2007. Pharmacological and clinical evaluation of L-asparaginase in the treatment of leukemia. Crit Rev Oncol Hematol 61, 208–221. https://doi.org/10.1016/j.critrevonc.2006.07.009

Poria, V., Rana, A., Kumari, A., Grewal, J., Pranaw, K., Singh, S., 2021. Current Perspectives on Chitinolytic Enzymes and Their Agro-Industrial Applications. Biology (Basel) 10, 1319. https://doi.org/10.3390/biology10121319

Rao, M.B., Tanksale, A.M., Ghatge, M.S., Deshpande, V.V., 1998. Molecular and Biotechnological Aspects of Microbial Proteases. Microbiol Mol Biol Rev 62, 597–635.

Rodriguez-R, L.M., Gunturu, S., Harvey, W.T., Rosselló-Mora, R., Tiedje, J.M., Cole, J.R., Konstantinidis, K.T., 2018. The Microbial Genomes Atlas (MiGA) webserver: taxonomic and gene diversity analysis of Archaea and Bacteria at the whole genome level. Nucleic Acids Res 46, W282–W288. https://doi.org/10.1093/nar/gky467

Sun, A., Song, W., Qiao, W., Chen, X., Liu, J., Luo, Q., Liu, L., 2017. Efficient agmatine production using an arginine decarboxylase with substrate-specific activity. Journal of Chemical Technology & Biotechnology 92, 2383–2391. https://doi.org/10.1002/jctb.5245

Sutto-Ortiz, P., Camacho-Ruiz, M. de los A., Kirchmayr, M.R., Camacho-Ruiz, R.M., Mateos-Díaz, J.C., Noiriel, A., Carrière, F., Abousalham, A., Rodríguez, J.A., 2017. Screening of phospholipase A activity and its production by new actinomycete strains cultivated by solid-state fermentation. PeerJ 5, e3524. https://doi.org/10.7717/peerj.3524

Verma, N., Kumar, K., Kaur, G., Anand, S., 2007. L-Asparaginase: A Promising Chemotherapeutic Agent. Critical Reviews in Biotechnology 27, 45–62. https://doi.org/10.1080/07388550601173926

Wang, L., Wang, S., Li, W., 2012. RSeQC: quality control of RNA-seq experiments. Bioinformatics 28, 2184–2185. https://doi.org/10.1093/bioinformatics/bts356

Wheelock, C.E., Shan, G., Ottea, J., 2005. Overview of Carboxylesterases and Their Role in the Metabolism of Insecticides. Journal of Pesticide Science 30, 75–83. https://doi.org/10.1584/jpestics.30.75

Wriston, J.C., 1985. [79] Asparaginase, in: Methods in Enzymology, Glutamate, Glutamine, Glutathione, and Related Compounds. Academic Press, pp. 608–618. https://doi.org/10.1016/S0076-6879(85)13082-X

Xu, W., Zhang, W., Zhang, T., Jiang, B., Mu, W., 2016. l-Rhamnose isomerase and its use for biotechnological production of rare sugars. Appl Microbiol Biotechnol 100, 2985–2992. https://doi.org/10.1007/s00253-016-7369-z
